# Genomic and transcriptomic analyses of the Chinese *Maotai*-flavored liquor yeast MT1 revealed its unique multi-carbon co-utilization

**DOI:** 10.1186/s12864-015-2263-0

**Published:** 2015-12-15

**Authors:** Xiaowei Lu, Qun Wu, Yan Zhang, Yan Xu

**Affiliations:** State Key Laboratory of Food Science and Technology; The Key Laboratory of Industrial Biotechnology, Ministry of Education; Synergetic Innovation Center of Food Safety and Nutrition; School of Biotechnology, Jiangnan University, Wuxi, Jiangsu China; Ministry of Education Key Laboratory of Systems Biomedicine, Shanghai Center for Systems Biomedicine, Shanghai Jiao Tong University, Shanghai, China

**Keywords:** *Saccharomyces cerevisiae*, Genome, Transcriptome, *Maotai*-flavour liquor

## Abstract

**Background:**

Revealing genetic mechanisms behind specific physiological characteristics of *Saccharomyces cerevisiae* from specific environments is important for industrial applications and requires precise understanding.

**Results:**

*Maotai* strain MT1 was isolated from the complicated Chinese *Maotai*-flavored liquor-making environment with extremely high temperatures, and acidic and ethanol stresses. Compared with the type strain S288c, MT1 can tolerate high acidity (pH 2.0), high ethanol levels (16 %) and high temperatures (44 °C). In addition, MT1 can simultaneously utilize various sugars, including glucose, sucrose, galactose, maltose, melibiose, trehalose, raffinose and turanose. Genomic comparisons identified a distinct MT1 genome, 0.5 Mb smaller than that of S288c. There are 145 MT1-specific genes that are not in S288c, including *MEL*1, *MAL*63, *KHR1*, *BIO1* and *BIO6*. A transcriptional comparison indicated that *HXT5* and *HXT13*, which are theoretically repressed by glucose, were no longer inhibited in MT1 and were highly expressed even in a medium containing 70 g/L glucose. Thus, other sugars may be co-utilized with glucose by MT1 without diauxic growth.

**Conclusions:**

Based on a functional genomics analysis, we revealed the genetic basis and evolutionary mechanisms underlying the traits of the Chinese *Maotai*-flavored yeast MT1. This work provides new insights for the genetic breeding of yeast and also enriches the genetic resources of yeast.

**Electronic supplementary material:**

The online version of this article (doi:10.1186/s12864-015-2263-0) contains supplementary material, which is available to authorized users.

## Background

The Chinese liquor-making process is a unique and complicated spontaneous fermentation process under solid state conditions [1]. Chinese liquor is produced by the technique of simultaneous saccharification and fermentation (SSF) using grains, such as sorghum, wheat and barley. The cost-effective SSF combines the enzymatic hydrolysis of starch with the simultaneous fermentation of sugars, resulting in an outpouring carbon source [2]. In addition, Chinese liquor is fermented using combinations of microorganisms, including yeast, bacteria and filamentous fungi [3]. The complex filamentous fungal community produces diversified hydrolases to degrade starch into various sugars, including glucose, galactose, maltose and melibiose [4]. High temperatures (~50 °C) at the starter are generated by the metabolic activities of the microorganisms and the low thermal conductivities of the solid matrices [5]. In addition, there are two other prominent pressures: acidity stress (pH 3.0) and ethanol stress (4.5–5.5 %, v/v) in the alcoholic fermentation stage [6]. Such a severe environment during the SSF process leads to a distinctive community of microorganisms with specific physiological properties and performances [7], in particular, distinctive heat- and acid-resistant properties [8].

Although the microorganisms in the Chinese *Maotai*-flavored liquor have been investigated since the 1960s [9], there is little information on the functional genomics of the yeast involved in the process. This has inhibited our understanding of the fermentation mechanism and the development of *Maotai*-flavored liquor. Functional genomics approaches, such as whole-genome sequencing, are powerful tools for the analysis of specific traits on a genomic scale and provide a method of studying cellular physiology from micro-perspective. Since the first complete genome of strain S288c was sequenced in 1996 [10], it has served as the reference for the *Saccharomyces cerevisiae* species and has promoted the development of whole-genome approaches [11]. Meanwhile, the genomes of several *S. cerevisiae* strains have been sequenced and compared with the S288c reference genome, including laboratory strains [12] and industrial strains, such as sake yeast K7, wine yeasts, beer yeasts and bioethanol yeasts [13–17]. The phenotypic diversity among yeast isolates is significant, and variation is apparent among the surveyed strains at different levels. Ecological factors and geographical locations, as the main reasons for biodiversity, have important effects on the domestication of microbial physiological characteristics [18]. Among the 50 strains with sequenced genomes in the NCBI database, there are only three strains from China [19]. However, strains involved in the SSF process of Chinese liquor are poorly understood. Theoretically, the genomic analysis of strains with different backgrounds should help identify the sequence changes that play important roles in specific physiological characteristics. Comparisons of the publicly available *S. cerevisiae* genome sequences have revealed the clear signatures [single nucleotide polymorphisms (SNPs), insertions and deletions (Indels), and novel ORFs] in the different strains [20]. Compared with laboratory strains, industrial strains generally show a higher adaptability to specific environments. However, the genetic basis for their improved characteristics is not well understood. Further studies are needed to explore how the genetic variations confer the specific phenotypes. The comprehensive identification of polymorphisms among individuals within a species is essential for studying the genetic basis of phenotypic differences and for elucidating the evolutionary history of the species.

In this study, *S. cerevisiae* MT1 was isolated from the fermentation environment of Chinese *Maotai*-flavored liquor. Its physiological characteristics were evaluated in several phenotypic assays. Meanwhile, the MT1 genome was sequenced and compared with the type strain S288c. In addition, we elucidated how genetic variations of MT1 were correlated with specified traits using RNA-seq and revealed the molecular mechanisms related to the adaptation of yeast in Chinese liquor-making. This whole-genome analysis of the *S. cerevisiae* strain used in Chinese liquor-making will supplement the genomic and phylogenetic knowledge of yeast and provide a guide for the construction of strains with desired traits.

## Results and discussion

### Phenotypic characterization

**Fig. 1:**
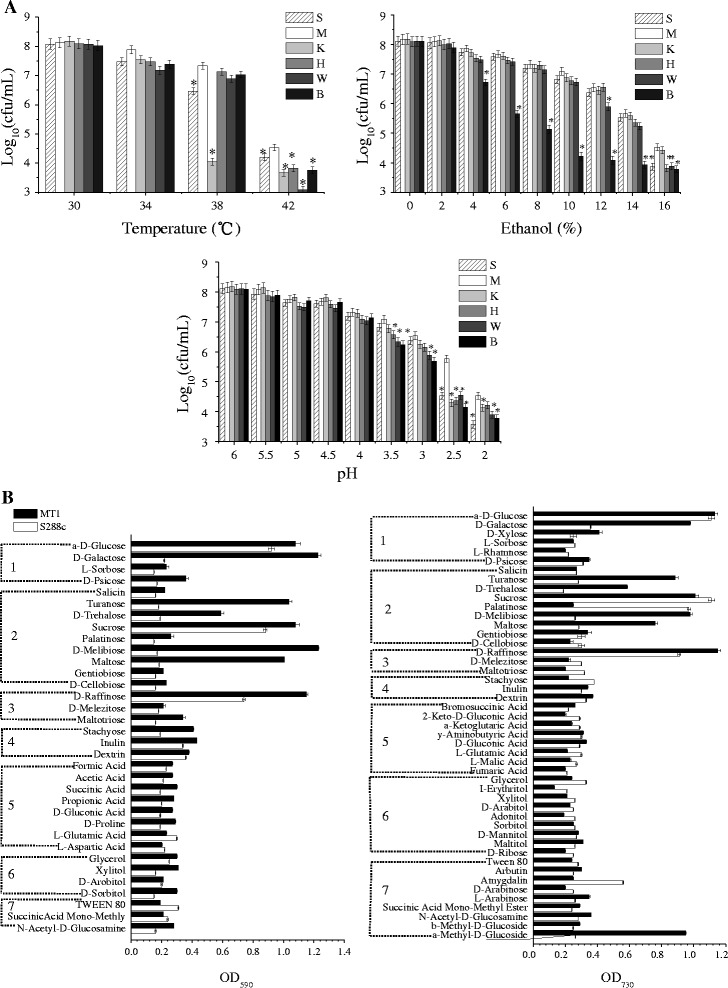
**a** Effects of temperature, ethanol and acidity on cell growth of several yest strains. S, *Saccharomyces cerevisiae* type strain S288c; M, Chinese *Maotai*-flavored liquor yeast MT1; K, Sake yeast strain K7; H, Hangjiu (Chinese Rice Wine) yeast CICC30394; W, Wine yeast strain CICC31089; B, Beer yeast strain ATCC9763. * represents the significant difference with *P*-value < 0.01, compared with MT1, calculated using a one-way analysis of variance in SPSS. **b** Growth differences between MT1 and S288c with various carbon sources. *Left*: oxidation tests that detect carbon utilization via a redox color change in the organism’s suspension; *Right*: assimilation reaction tests that are based on turbidity increases due to carbon utilization of the organisms. 1, monosaccharides; (2), disaccharides; (3), trisaccharides; (4), oligosaccharides; (5), acids; (6), alcohols; and (7), others

Several physiological and biochemical characteristics of the *S. cerevisiae* strain MT1, isolated during the process Chinese *Maotai*-flavored liquor-making, were compared with those of the reference strain S288c and other industrial strains (Fig. 1a). In addition to the sake yeast K7, other strains had high tolerance to 38 °C which is consistent with previous studies that it was widely believed that *S. cerevisiae* had a thermal tolerance of 25–37 °C or 39 °C [21]. While a higher thermal resistance was found for MT1 when temperature was higher than 38 °C compared with not only S288c but also other industrial yeasts. Population of MT1 was twice that of other strains (besides the sake yeast K7) in 16 % ethanol. It indicated that MT1 had a higher ethanol tolerance. In addition, MT1 had a growth advantage compared with other strains at pH 3.5–2.0. In particular, the population of MT1 is almost three times of that of S288c at pH 2.0. This suggested that MT1 has a much higher resistance to acidity than S288c and other industrial strains, as most industrial strains could barely grow at pH 2.5 [22].

Carbon source utilization exerts a strong influence on the classification of isolates and may be a useful and functional measure for the characterization and classification of different strains. Unfortunately, there is limited research on the simultaneous fermentation of various carbon sources to produce ethanol. The primary reason is that not many strains can ferment ethanol from various sugars. Presently, there is some research on a strain that could simultaneous utilize glucose and xylose, or arabinose and galactose [23]. MT1s ability to uptake and assimilate sugars was also investigated (Fig. 1b). Among the monosaccharides, glucose could be used for growth and fermentation by both MT1 and S288c, with OD_590_ and OD_730_ values exceeding 0.8. When galactose was the sole carbon source, the OD_590_ and OD_730_ of MT1 reached 1.23 and 0.98, respectively, while those of S288c were less than 0.3. This indicated that MT1 could uniquely utilize galactose. The other carbon sources that MT1 could uniquely uptake and assimilate were mainly disaccharides, such as melibiose, maltose, trehalose and turhanose. In addition, raffinose, a trisaccharide constituted by galactose, fructose and glucose, as well as methyl-a-D-glucopyranoside, are well utilized by MT1, with OD_730_ values reaching 1.15 and 0.95, respectively. These results suggested that MT1 was a strain appropriate for multi-carbon fermentation, having the ability to utilize various carbon sources.

### MT1 genome structure

The MT1 genome assembly resulted in 125 contigs (>200 bp) with an N50 value of 390,837. Then, 89 scaffolds (>500 bp) were obtained with an N50 value of 550,408. The assembled data were evaluated, and the detailed statistical data are shown in Table 1. Finally, the MT1 assembled genome was 11.62 Mbp with a GC% of 38.08 %. The genome size of MT1 is smaller than most of the *S. cerevisiae* genomes deposited in the NCBI database (Table 2). A total of 5,106 genes were predicted with an average length of 1.6 kb, occupying 69.26 % of the whole genome (Table 2).

**Table 1:** Chinese Maotai-flavored liquor yeast MT1 library assembly statistics

	Contigs (>200 bp)	Scaffolds (>500 bp)
Min sequence length (bp)	205	508
Max sequence length (bp)	899,603	1,037,495
Total sequence number	125	89
N20	749,375	805,328
N50^a^	390,837	550,408
N90	128,295	233,221
N number	0	26946
N rate %	0	0.23 %
Total sequence length (bp)	11,620,879	11,620,879
GC content %	38.08 %	38.08 %
Sequences greater than 1 kb	73	44

N20 and N90 were obtained in the same way

^a^N50: computed by sorting all contigs from largest to smallest and by determining the minimum set of contigs whose sizes total 50 % of the entire genome

**Table 2:** Essential *Saccharomyces cerevisiae* genomic information

	MT1	S288c	K7	AWRI1631	YJM789
Genome size (Mb)	11,62	12,16	12,39	11,18	11,99
GC content (%)	38.08	38.15	38.30	38.20	38.10
GC content in mRNA region (%)	39.62	/	/	/	/
rRNA number	5	10	1	/	/
tRNA number	312	299	296	/	/
Coding sequence number	5,106	5,906	5,816	5,451	5,912
Average coding sequence length (bp)	1,576	1,369	/	/	/
Total coding sequence length (Mb)	8,05	9,16	/	/	/
Coding sequence of genome (%)	69.26	75.37	/	/	/

**Fig. 2:**
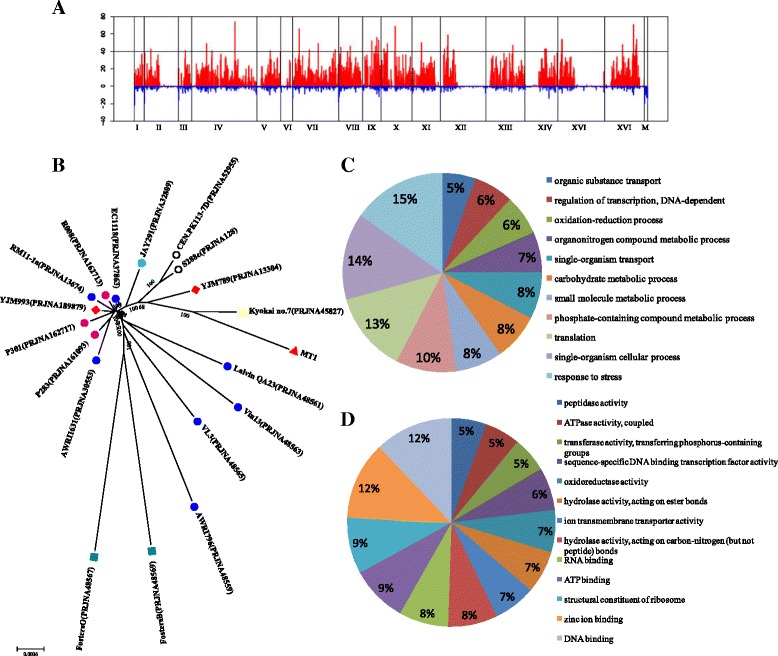
**a** Genome-wide distribution of heterozygosity in the Chinese *Maotai*-flavored liquor yeast MT1 genome. The frequency of the extracted heterozygosity was plotted using 10-kb windows of the chromosomal coordinates, where *black vertical lines* represent the division between two chromosomes. Heterozygous SNPs or Indels in MT1 are shown in *red* and *blue*, respectively. **b** Phylogenetic tree generated by Mega5 [51] using the whole genome sequence of MT1 and the available *Saccharomyces cerevisiae* genomes in NCBI. Different strains are represented in the diagrams by different shapes and colors. *Red triangles*, the typical Chinese liquor fermentation strain MT1; *yellow triangles*, sake yeast strain; red rhombuses, clinical strains; *white circles*, library strains; *aqua circles*, bioethanol strain; *blue circles*, wine strains from Europe; *pink circles*, wine strains from Italy; and *dark cyan circles*, beer strains. **c** Functional classification of the specific genes in MT1. **d** Functional classification of the genes lost from MT1

Within the aligned regions of the MT1 and S288c genomes, we identified 58,960 high-confidence SNPs (44,847 homozygous SNPs and 14,113 heterozygous SNPs, Additional file 1: Table S1), including 13,780 missense variants and 25,724 synonymous variants (Additional file 1: Table S2). Remarkably, the level of nucleotide polymorphism observed between MT1 and S288c is very similar to that of YJM789 (60,339), a strain of *S. cerevisiae* isolated from the lung of an AIDS patient, and the wine yeast strain AWRI1631 (56,703) [15], far higher than the biofuel producing industrial strain YJS329 (39,098) which was also isolated from China [19] and the commercial wine yeast strain EC1118 (46,152) [16], but far lower than that of the sake yeast strain K7 (67,725) [13], and the biofuel producing industrial strain JAY291 (65,000) [17]. In addition, there were 6,474 small Indels (<90 bp), including 4,457 homozygous Indels and 2,017 heterozygous Indels (Additional file 1: Table S1). Within the annotated Indels, there were 251 frameshift variants that resulted in feature elongation, and 262 frameshift variants that resulted in feature truncation (Additional file 1: Table S3). The densities of SNPs and Indels were far from constant across either the whole genome (Additional file 1: Table S1) or individual chromosomes (Fig. 2a). This phenomenon mostly appears in genomes of industrial strains, such as the genome of K7, AWRI1631 and YJS329. It is more likely that a complex history of out- and/or back-crossings of the ancestral strain in the industrial environments by environmental domestication and artificial breeding could have caused this pattern. In addition, 183 copy number variations were obtained using CNVnator [24]. They were comprised of 30 duplication regions and 153 deletion regions (Additional file 1: Table S4). All of these results indicated that MT1 had a genome distinct from that of S288c. Aimed at detecting the evolutionary origin of MT1, a neighbor-joining tree was constructed on the basis of whole genome and protein sequences. The tree included MT1 and 18 other representative *S. cerevisiae* strains from library, clinic and sake, bioethanol, wine and beer production. As shown in Fig. 2b, MT1 is located far from the wine and beer strain clusters but adjacent to the sake yeast strain K7, and they were both isolated in Asia.

Among the 5,106 genes of MT1, less than 500 genes displayed similarity levels below 95 % when compared with S288c (Additional file 1: Figure S1). Using an all-vs-all BLASTP and clustering analysis with orthomcl (V2.0.8), 145 MT1-specific genes were identified, 90 % of which had near-perfect-match hits to the nucleotide sequence of S288c, and likely reflect ORF annotation differences, whereas others had nonreciprocal-best-hit homologs in S288c. The functions of these genes fell within stress response (14 genes), carbohydrate metabolic process (seven genes), organic substance transport (five genes), biosynthetic process (24 genes), organic substance metabolic process (42 genes), and others (Fig. 2c). In addition, 695 genes were missing from MT1 compared with S288c. Among them, there were 25 genes and 21 genes in the location of centromere and telomeres, respectively, where assemble errors happen frequently. These genes are *Mic17p*, *VMA9*, *TAF11*, *TIR4*, *TIR2*, *PRP18*, *COX16*, *COX17*, *SHU1*, *MNN1*, *RFA1*, *PUF4*, *AVT1*, *TEN1*, *GAT3*, *CMS1*, *GAT3*, *SIN3*, *PFA4*, *MCD1*, *PSF1*, *PDR3*, *RER1*, *SGF29, PAU24, PAU12, PAU14, PAU1, PAU18, PAU6, PAU21, PAU22, PAU13, PAU9, PAU3, PAU15, PAU16, PAU20, PAU10, RDS1, HSP32, YPS5, AIF1, BDS1* and *ENB1.* 60 % of 695 missing genes were hypothetical or uncharacterized ORFs encoding putative proteins of unknown functions as reported in the Saccharomyces Genome Database. The other 40 % of the genes were mainly involved in ion transport, regulation of transcription and translation, DNA and RNA binding and cellular component synthesis, such as membrane components, organelle organization and cellular protein components (Fig. 2d). While there were only about 10 genes listed in S288c as essential, including *DAD2*, *DAD3*, *DAD4*, *BRR6*, *RRP14*, *ESF2*, *FAP7*, *USO1*, *MCD1* and *SPC19*. Function of each genes was listed in Additional file 1: Table S5. As indicated by the functional classification of the specific and lost genes in MT1, there is a speculation that MT1 may have a special genomic system. It weakened transcriptional, translational and cytoplasmic synthesis. On the other hand, it strengthened carbohydrate transport and metabolism, energy metabolism and informational processing (especially environmental informational processing) metabolism.

### Transcriptomic comparison using RNA-seq

The transcriptomic comparison between MT1 and S288c was carried out in sorghum extract (pH 6.0), which is the raw material for liquor-making. The expression levels of genes related to carbohydrate and energy metabolism were significantly up-regulated in MT1 compared with in S288c (Table 3). Within the carbohydrate metabolism, the most significant responsive pathway was the tricarboxylic acid cycle (ko00020) with 12 up-regulated genes. In addition, five genes in the pentose phosphate pathway (ko00030) and four genes in galactose metabolism (ko00052) were more highly expressed than in S288c. In energy metabolism, oxidative phosphorylation (ko00190) had the largest number of up-regulated genes. Nucleotide metabolism, translation, and cell growth and death were the responsive pathways that had a large number of down-regulated genes compared with in S288c (Table 2). Ribosome (ko03010) had the largest number of markedly lower expressed genes (84 genes). Based on the analyses of the transcriptome, we predicted that, compared with other yeast strains, MT1 tends to make higher investments in carbon and energy metabolism than transcription and translation. This is consistent with the corresponding speculation indicated by the functional classification of the specific and lost genes in MT1.

**Table 3:** Main differences in the metabolic pathways of the Chinese *Maotai*-flavored liquor yeast MT1 compared with the *Saccharomyces cerevisiae* type strain S288c when grown in Sorghum juice medium, pH 6.0

Metabolism	-Log10 (*P*-value)	Pathway	Up genes	Down genes
Carbohydrate metabolism	16.82	Tricarboxylic acid cycle cycle (ko00020)	12	1
		Pentose phosphate pathway (ko00030)	5	3
		Galactose metabolism (ko00052)	4	2
		Butanoate metabolism (ko00650)	3	1
Energy metabolism	8.34	Oxidative phosphorylation (ko00190)	18	1
		Sulfur metabolism (ko00920)	2	0
		Carbon fixation pathways (ko00720)	3	1
		Sulfur metabolism (ko00920)	2	0
Nucleotide Metabolism	1.97	Purine metabolism (ko00230)	0	16
		Pyrimidine metabolism (ko00240)	1	13
Translation	29.25	Ribosome (ko03010)	0	84
		Aminoacyl-tRNA biosynthesis (ko00970)	0	9
		RNA transport (ko03013)	0	9
		Ribosome biogenesis in eukaryotes (ko03008)	0	25
		RNA degradation (ko03018)	0	6
Cell growth and death	1.70	Cell cycle (ko04110)	0	6
		Cell cycle – yeast (ko04111)	0	21
		Meiosis – yeast (ko04113)	4	16

**Fig. 3:**
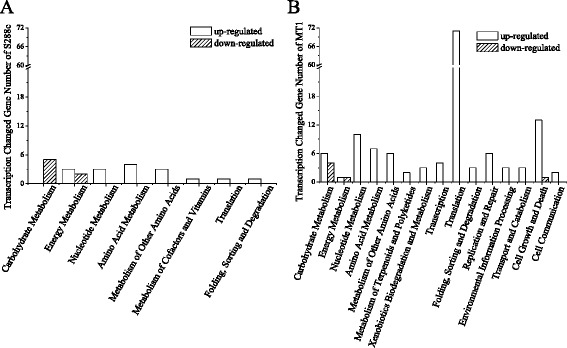
Transcriptionally changed pathways of the *Saccharomyces cerevisiae* type strain S288c (**a**) and the Chinese *Maotai*-flavored liquor yeast MT1 (**b**) when the pH of the sorghum juice medium was reduced to 2.7 from 6.0

Although there was no difference between the growth of MT1 and S288c at pH 6.0, MT1 had a gradually increasing growth advantage as the pH decreased to 2.0 (Fig. 1a). S288c could not grow when the pH was below 2.5, thus a pH level of 2.7 was selected as the control condition, versus pH 6.0 for transcriptomic comparisons, to identify the genetic mechanisms of acid tolerance. Then, the two strains were compared at pH levels between 6.0 and 2.7. When the pH was reduced to 2.7 from 6.0, S288c genes in seven pathways were up-regulated (Fig. 3a), including nucleotide metabolism (four genes), amino acid metabolism (four genes), translation (one gene) and folding, sorting and degradation (one gene). Within energy metabolism in S288c, three genes were up-regulated, while two genes were down-regulated. In addition, five carbohydrate metabolism genes were down-regulated. However, there were 15 responsive pathways in the MT1 transcriptome, indicating that MT1 had a stronger response to acid than S288c. The seven responsive pathways in S288c were also identified in MT1 but involved many more responsive genes. In particular, the translational pathway in MT1 had 71 up-regulated genes. The other responsive pathways that had up-regulated genes in MT1 were involved in transcription, replication and repair, environmental information processing, transport and catabolism, cell growth and death, and cell communication (Fig. 3b). In addition, several metabolic pathways of MT1 had genes up-regulated, such as terpenoid and polyketide metabolism, xenobiotic biodegradation and metabolism, and carbohydrate metabolism. In particular, terpenoids and polyketides are the important healthy active materials in Chinese liquor. As indicated by the transcriptomic comparison, the transcription- and translation-related genes of MT1, which were inactive compared with those of S288c under normal conditions, were more active under acidic condition and achieved the same level of expression as in S288c.

Under normal conditions (sorghum juice medium, pH 6.0), there were not only less genes related to transcription and translation in MT1 but they were also expressed at far lower levels than in S288c; however, MT1 could attain a greater biomass than S288c. When the pH was reduced from 6.0 to 2.7, genes with functions related to transcription, replication and repair and, especially, translation in MT1 were strongly up-regulated. Within these, several genes are essential to acid pH resistance, such as *CIK1*, *HSP30*, *RPS27B*, *RPL31A*, *RPL18* and *ARO2.* There may be two reasons for this strange phenomenon. One is that MT1 requires less proteins and nucleotides for growth than S288c. The other may be that genes involved in transcription, translation, replication and repair in MT1 are more efficient than those in S288c. There is no need to mobilize a large number of related genes under suitable conditions. Once threatened, the cell will increase its investment to survive.

### Genetic mechanism of multi-carbon co-utilization

**Fig. 4:**
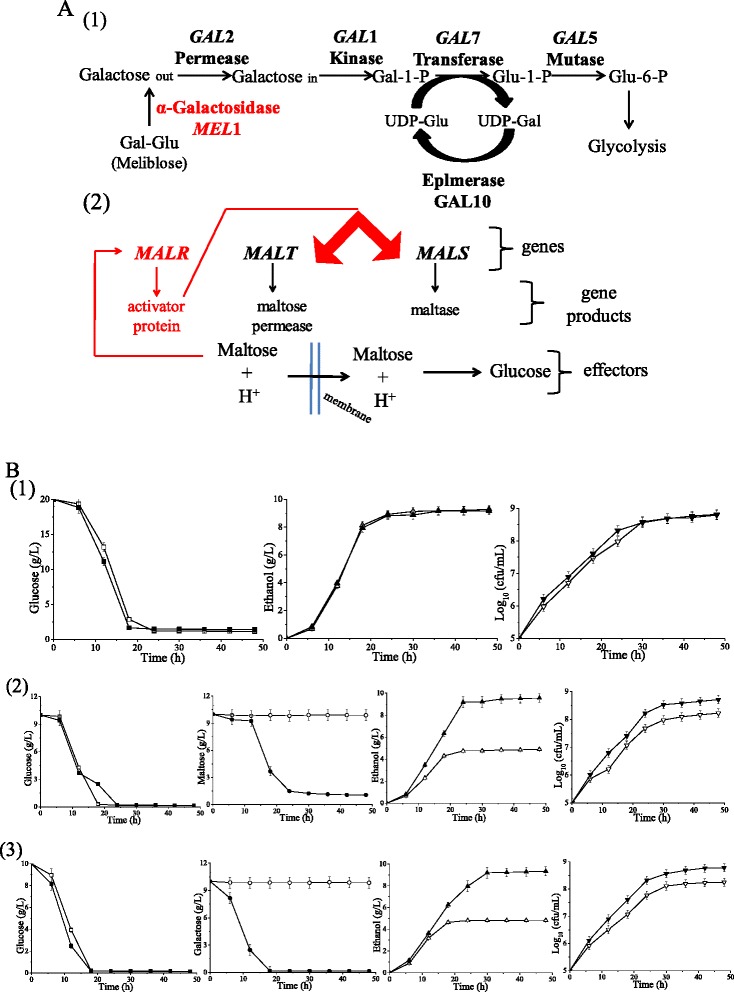
**a** Metabolic pathways and gene regulatory mechanisms of galactose, melibiose and maltose. The red parts of the pathways are specific to the Chinese *Maotai*-flavored liquor yeast MT1 strain. **b** Dynamic curves of fermentation parameters. (1) YPD medium (1 % yeast extract, 2 % peptone and 2 % glucose); (2) modified YPD medium (1 % yeast extract, 2 % peptone, 1 % glucose and 1 % maltose); (3) modified YPD medium (10 % yeast extract, 20 % peptone, 1 % glucose and 1 % galactose). The open figures represent the *Saccharomyces cerevisiae* type strain S288c; the solid figures represent MT1

MT1 can uniquely assimilate several disaccharides, such as melibiose and maltose, and some monosaccharides, such as galactose. However, the MT1 and S288c contain the same genes related to galactose utilization and its regulatory mechanism, including *GAL*2, *GAL*1, *GAL*7, *GAL*5, *GAL*10 and others (Fig. 4a). Therefore, a transcriptomic comparison was carried out between the two strains. It was discovered that several genes involved in galactose metabolism, such as *YHR104w*, *YIL162W*, *IMA5* and others, in MT1 were up-regulated compared with those in S288c. The enzyme of *YHR104w*, *akr1* (EC:1.1.1.21), is responsible for the transformation between D-galactose and galactitol, while *YIL162W* encodes the enzyme *sacA* (EC:3.2.1.26) which is involved in the degradation of raffinose into melibiose or galactose. The metabolic networks of melibiose and galactose are only different in the initial step, the intracellular breakdown of melibiose into glucose and galactose [25]. This step involves an alpha-galactosidase encoded by *MEL1*. This important gene was identified in contig 8 of MT1 but does not exist in S288c. It is most likely the reason for MT1’s specific ability to utilize melibiose. This gene was identified in the genome of the laboratory strain, *S. cerevisiae* CEN.PK113-7D [26], a model strain for industrial biotechnology. The enzyme system of maltose is a typical inducible system in yeast cells, which includes the maltose permease and maltase [27]. The *MAL* loci is an important gene in maltose metabolism and is composed of three genes: *MALS*, *MALT* and *MALR* (Fig. 4a). The *MALR* genes encode maltose fermentation regulatory proteins that induce the expression of *MALS*, encoding the maltase (alpha-D-glucosidase), and *MALT*, encoding a high-affinity maltose transporter, maltose permease [28]. These three kinds of genes were identified in the MT1 genome. The two *MALT* genes (*MAL11* and *MAL31*) and one *MALS* gene (*MAL32*) also exist and are functional in the genomic reference strain S288c. The differences mainly exist in the regulatory protein encoding genes: *MAL13*, *MAL33* and *MAL63*. Notably, *MAL63* is absent in the reference strain S288c, while *MAL13* and *MAL33* exist but are non-functional. In addition, there were no differences in the expression levels of *MAL13* and *MAL33* between MT1 and S288c. This suggests that *MAL63* might be the important gene of the MT1’s maltose metabolism pathway. In contrast to S288c, strains from the CEN.PK lineage are able to grow on maltose when it is the sole carbon source. However, unlike MT1, the *MAL23* mutant allele known as *MAL2-8C* is responsible for the utilization of maltose in CEN.PK strains [26].

The transport of sugars across the cell membrane is important for growth and fermentation. Our analyses suggest that MT1 possesses different sugar uptake and assimilation properties than S288c. MT1 contains 17 HXT family genes, encoding hexose transporter proteins. Among these genes, *HXT4*, *HXT7*, *HXT9*, *HXT12*, *HXT13*, *HXT16* and *HXT17* in the MT1 genome were identified by local BLAST searches and confirmed by PCR testing. The products of these genes show different glucose affinities and different expression levels to coordinately control glucose uptake in environments containing a broad range of glucose concentrations [29]. When cells were collected for a transcriptomic analysis, we found that the expression levels of *HXT5* and *HXT13* in MT1 were much higher than in S288c, even though the glucose concentration in the sorghum extract adopted for the transcriptomic assay reached 70 g/L (Table 4). This differs from a conventional report [30] that suggested that the two genes were repressed by high levels of glucose. In particular, as the glucose concentration rose from 0.2 to 40 %, the expression level of *HXT*13 decreased by 80 %. The high expression levels of *HXT5* and *HXT13* indicate that MT1 may have no glucose repression and is able to simultaneously utilize multiple sugars.

**Table 4:** Comparison of the expression levels of *HXT* genes between the Chinese *Maotai*-flavored liquor yeast MT1 and the *Saccharomyces cerevisiae* type strain S288c when grown in sorghum juice medium (70 g/L glucose)

Gene name	Gene function	BaseMeanMT1	BaseMeanS288c	*P-*value
*HXT1*	Low-affinity glucose transporter induced by Hxk2p in the presence of glucose and repressed by Rgt1p when glucose is limiting.	18.18	238.50	1.11 × 10^−7^
*HXT2*	High-affinity glucose transporter induced in low glucose and repressed in high glucose.	2523.13	73238.62	2.66 × 10^−4^
*HXT 3*	Low affinity glucose transporter induced in low or high glucose conditions.	770.75	186705.11	2.02 × 10^−7^
*HXT5*	Hexose transporter with moderate affinity for glucose induced in the presence of non-fermentable carbon sources and induced by a decrease in growth rate.	191488.64	67393.35	1.96 × 10^−1^
*HXT8*	Protein of unknown function similar to hexose transporters, expression is induced in low glucose and repressed in high glucose.	20.60	62.17	4.64 × 10^−1^
*HXT9*	Putative hexose transporter similar to major facilitator superfamily (MFS) transporters, expression is regulated by transcription factors Pdr1p and Pdr3p.	163.60	193.65	8.69 × 10^−1^
*HXT10*	Putative hexose transporter, expressed at low levels and expression is repressed by glucose.	3.64	18.35	6.44 × 10^−1^
*HXT13*	Hexose transporter, induced in the presence of non-fermentable carbon sources, induced in low glucose and repressed in high glucose.	124014.98	588.09	3.34 × 10^−7^
*HXT14*	Protein similar to hexose transporter family members, expression is induced in low glucose and repressed in high glucose	9.70	12.23	9.70 × 10^−1^

To verify the multi-carbon co-utilization without glucose repression, which was indicated by the genomic and transcriptomic analyses, cell growth, sugar consumption and ethanol production were compared between MT1 and S288c in media containing mixed sugars. As shown in Fig. 4b, when compared with S288c in 2 % glucose, MT1 grew faster but showed no difference in the glucose consumption and ethanol production. However, in 1 % glucose plus 1 % maltose, MT1 could use maltose to grow and ferment, and consequently its biomass and ethanol yield were twice that of S288c. This was also true in 1 % glucose plus 1 % galactose. As indicated by the dynamic curves of sugar consumption (Fig. 4b), MT1 could utilize maltose and galactose in the early stages of the logarithmic phase without the phenomenon of secondary growth. This was confirmed by the lack of a “lag” period in the growth of MT1 in a medium containing mixed sugars (Fig. 4b). These results indicate that the high expression levels of *HXT5* and *HXT13* without glucose repression may contribute to the multi-carbon co-utilization.

### Other unique genes lead to unique phenotypes

*KHR1* is another unique gene of MT1, encoding a heat-resistant killer toxin. This gene is located in a 2-kb fragment inserted into MT1 scaffold 22, which can be mapped to S288c chromosome IX. This ORF has been identified at the same location in the genome of YJM789 [31]. The killer toxin provides MT1 with a stronger survival rate compared with other microorganisms in the Chinese liquor fermentation environment. In addition, two specific *BIO* genes were found on contig40, resulting in MT1 having biotin prototrophy. The two *BIO* genes share 100 % identity with *BIO1* and *BIO6*, which encode pimeloyl-CoA synthase and KAPA synthase [32], respectively. They are also conserved (over 99 %) in several other *S. cerevisiae* strains, such as Sigma1278b (GenBank: ACVY00000000.1), YJM269 and UC5 (GenBank: AFDD00000000.1). As shown in Additional file 1: Figure S2, *BIO1* is located between *ESMT1* and *MCM10*, while *BIO6* lies between *AXL2* and *REV7*. Compared with S288c, MT1 could grow in the medium without biotin (data notshown). This confirmed the biotin prototroph of MT1.

## Conclusions

The physiological and biochemical characteristic diversity of microbes is very important for industrial applications. Our analysis indicated that MT1 not only had higher tolerances to acidity and ethanol but was also capable of simultaneously producing ethanol from various carbon sources without glucose repression. These abilities could be applied to cellulose ethanol to eliminate the bottleneck during multi-carbon source fermentation. In addition, MT1 is a biotin prototroph, a phenotypic trait with potential in industrial applications. Biotin (vitamin H) is an essential nutrient for all organisms, being a cofactor of many enzymes involved in carboxylation reactions, such as gluconeogenesis, amino acid metabolism, fatty acid biosynthesis and energy metabolism [33]. These physiological and biochemical characteristics place MT1 in a special category of yeast desirable for industry. Revealing the genetic basis of these traits would provide a platform for not only understanding Chinese liquor but also for developing an economically advanced biofuel process. It is necessary to analyze the evolutionary mechanisms of such special phenotypic and physiological characteristics. We assume that these variances may be induced by the raw materials and environmental stresses in the process of Chinese liquor-making. The SSF technique used in making Chinese liquor combines the enzymatic hydrolysis of starch with the simultaneous fermentation of sugars. Diverse hydrolases produced by the complex filamentous fungal community generate various sugars. This results in various carbohydrates that can be utilized by cells to grow and produce ethanol. In addition, Chinese *Maotai*-flavored liquor is produced by a complicated SSF process in an extremely severe environment, with high temperatures, as well as acidic and ethanol stresses. In the environment, temperatures could reach 50 °C, the pH could be as low as 3.5, and the ethanol concentration could be as high as 5.5 %. Over time, this specific environment has produced specific physiological properties and performances.

Environmental pressure is the external induction force, while the specific genome contains the internal underlying determinants. The genomic structural analysis indicated that MT1 retained a smaller genome than the reference strain S288c and some other industrial strains (Table 1). The genome of MT1 has lost several segments mostly comprised of non-coding protein genes, such as the large missing fragment of 25 kb on Chromosome I (Additional file 1: Figure S3A), which was verified by PCR (Additional file 1: Figure S3B). In S288c, this segment contains only one functional gene, *FLO*9. MT1 also lost hundreds of genes whose functions fell within transcription, translation, DNA and RNA binding, and cellular component synthesis. In addition, MT1 gained several specific genes, such as *MEL1* and *MAL63*, related to its multi-carbon co-utilization, and *KHR*1, associated with its competitiveness. These genomic adaptations embody the economy of the MT1 strain: removing some redundant genes, retaining necessary genes for growth and fermentation, and adapting to the complex fermentation environment of Chinese liquor. Consequently, these alterations make MT1 a naturally ascendant strain with a minimal genome, containing special functional genes/clusters [34]. The small genome has a higher economic efficiency. It may have been beneficial to reduce the metabolic redundancy and then improve the metabolic efficiency [35]. Therefore, MT1 has the advantages of a short growth cycle, high production efficiency, high conversion rate, and strain stability in the fermentation process.

Various mechanisms are known to be involved in the adaptive evolution of yeast to the fermentation process, such as gene duplication, polyploidy, chromosomal rearrangements, interspecific hybridization and introgression [36]. On the basis of diverse examples drawn from both mammalian and microbial genetics, Maynard V. Olson proposed the “less is more” hypothesis that states that genetic loss may be an important engine of evolutionary change [37]. Aravind et al. [38] and Braun et al. [39] revealed that *S. cerevisiae* may have lost at least 300 genes, and another 300 or so genes have become highly diverged, since their split from their common ancestor with *Saccharomyces pombe*. A notable feature of the lost genes is the co-elimination of functionally connected groups of proteins, such as the signalosome and the spliceosome components. The other missing genes are involved in basic cellular processes, translation and ion homeostasis. However, intriguingly, some of the strains in which genes had been disrupted grew better than wild-type strains on a rich medium [40]. The counterintuitive idea that “less is more”, is well-reflected in the evolution of MT1. The gene loss in MT1 has resulted in no obvious disadvantages in growth and fermentation, but provide MT1 the advantages of a short growth cycle and strain stability. Furthermore, a speculative hypothesis is proposed that MT1 gained the genomic space to obtain special functional genes (such as *MEL1*, *MAL63*, *BIO1*, *BIO6* and *KHR1*) by reducing the gene redundancy. This strategy of evolution by gene loss is an important aspect of yeast diversification and may play a major role in their adaptation to the Chinese liquor fermentation ecosystem.

By revealing the genetic basis of specific phenotypes and elucidating the evolutionary history of the Chinese *Maotai*-flavored liquor yeast MT1, this work enriches our knowledge of *S. cerevisiae,* especially with regard to minimal genomic research. Furthermore, this functional genomics study of industrial microorganisms could be beneficial in improving breeding strategies to obtain the desired production traits in industrial microorganisms.

## Methods

### Yeast strains and culture conditions

*S. cerevisiae* S288c and sake yeast Kyokai No. 7 (K7) were obtained from the National Institute of Technology and Evaluation. Diploid strain MT1 was isolated from samples of the *Maotai*-flavored liquor production distillery in Guizhou, China. Strains from wine, beer or Chinese Rice Wine (Huangjiu) were obtained from China Center of Industrial Culture Collection (CICC). The yeast extract peptone dextrose (YPD) growth medium contained 10 g/L yeast extract, 20 g/L peptone and 20 g/L glucose.

### Analysis of yeast tolerance to several stress conditions

Three stress factors that are important features in liquor fermentation were studied: temperature, pH, and ethanol concentration. Except for during the temperature stress test, all cultures were incubated at 30 °C. For each stress factor, several fermentation conditions were tested, including four temperatures (30, 34, 38 and 42 °C), a range of pH levels (2.0 to 5.5) and several ethanol concentrations (0 to 16 %). Yeast was precultured in YPD medium at 30 °C for 24 h. Cells were diluted in sterile water and then was cultured in stress media under static cultivation at 30 °C for 48 h with the same inoculum concentration of 1 × 104 cfu/mL.

### Carbon source assimilation experiment

Biolog YT MicroPlates were used to test the ability of MT1 and S288c to utilize or oxidize compounds from a preselected panel of different carbon sources. The test yields a characteristic pattern of purple wells, which constitutes a “Metabolic Fingerprint”. The isolate to be identified is grown on an agar medium and then suspended in sterile water. Then, the cell suspension is inoculated into the YT MicroPlate, at 100 μl per well. All of the wells are colorless when inoculated. During the incubation there is a burst of respiration in the wells, which contain chemicals that can be oxidized, and the cells reduce the tetrazolium dye, forming a purple color. Meanwhile, microbes grow in the wells that contain utilizable carbon sources, leading to a higher turbidity. Negative wells remain colorless, as do the reference wells (A1 and D1) that contain no carbon sources. The OD_590_ values of lines A–C and the OD_730_ values of lines D–H were detected after 48 h of cultivation at 30 °C. These values indicated that the carbon source could not be utilized when the corresponding OD_590_ or OD_730_ was below 0.2, but could be at values from 0.2 to 0.8, with values >0.8 having the best carbon utilization rates.

### Preparation of the sorghum extract medium

To prepare the sorghum extract medium, 2 kg milled sorghum powder was added to 8 L deionized water. The mixture was steamed for 2 h and then saccharified by glucoamylase (5 U/L) at 60 °C for 4 h. The supernatant was collected after being filtered through gauze and centrifuged at 8,000 × *g* for 15 min. The sugar content was measured on a Leica refractometer (Fisher Scientific, Pittsburg, PA, USA). Apply few solution on the scope then gently close the cover plate. Turn the eyepiece to read the value by adjusting the handwheel. The mixture was then diluted with water to produce a final sugar concentration of 10° Brix (80 ± 5 g/L reducing sugar) before sterilization.

### Sequencing and assembly

The MT1 genome was sequenced using the whole-genome shotgun sequencing approach in the Illumina Miseq platform [41], and four paired-end/mate-paired sequencing libraries were constructed with insert sizes of 450 bp, 700 bp, 3 kb and 8 kb. Raw data were assembled into contigs and scaffolds employing the *de novo* assembler Newbler [42] and SSPACE [43]. The assembled sequences were manually checked, and the gaps were closed using the GapCloser program [44]. The sequences of the final contigs were deposited in GenBank under the Whole Genome Shotgun project [GenBank: JRUS00000000].

### Gene prediction and annotation

We predicted the MT1 genome using the MAKER pipeline prediction system, combining Augustus [45], SNAP [46] and the Glimmer gene prediction software [47]. The functional annotation was based on GO and KEGG [48] databases. Orthology with the S288c ORFs was evaluated by employing BLASTP similarities.

### Sequence comparison and SNPs/Indels analysis

An overall comparison of the MT1 genome sequence with 16 chromosome sequences from S288c was performed using BLASTN. SNPs and Indels based on strain S288c were estimated using the GATK pipeline system and Unified Genotyper, and then they were annotated using Variant Effect Predictor provided by Ensembl.

### Construction of the evolutionary tree

First, a family containing 18 yeast genomes was created using the software Orthomcl to obtain the protein sequences with a strict 1:1:1 ratio. Then, the protein sequences were compared using Muscle (version 3.8.31) software with the default parameters. Unreliable sequence alignment loci were removed using Gblock (version 0.91 b). Finally, the phylogenetic tree was constructed by Mega5 software using the neighbor-joining method [18], Poisson model and uniform rates. We also used the bootstrap method to validate the reliability of the evolutionary tree branches with 1,000 bootstrap replications.

### cDNA preparation and transcriptomic analysis using RNA-seq

Genomic DNA was digested using DNase, and total RNA was isolated using the TRIzol reagent. The OligoTex mRNA mini kit (Qiagen) was used to purify poly(A) mRNA from the total RNA samples. Then, the mRNA was fragmented using chemical reagents and high temperatures, and the DNA was synthesized, purified and enriched. The sequencing of the cDNA library products was performed on an Illumina HiSeq 2000 platform. After removing reads containing sequencing adapters and reads of low quality [reads in which the percentage of low quality bases (quality value ≤5) was more than 50 %], the remaining clear reads were aligned to *S. cerevisiae* S288c or MT1 genes using bowtie2/tophat2. The expression levels were normalized by reads per kilobase of exon region per million mapped reads (RPKM) [49]. The screening of differentially expressed genes and *P*-value calculations were performed with the method of DESeq [50]. Three biological replicates were performed for each time point for each strain.

## Additional file

Additional file 1: Table S1. Statistics of the SNPs and Indels among the MT1 Chromosomes. **Table S2.** Annotation of SNPs. **Table S3.** Annotation of Indels. **Table S4.** Copy Number Variations (CNVs) between MT1 and S288c. **Table S5.** Functions of the missed genes. **Figure S1.** Statistical analysis on the number of contigs with identity <95 % in the amino acid levels. **Figure S2.** Analysis of two *BIO* genes present in the LI genome, but not in the S288c genome. A: Schematic organization of two relevant segments from MT1-contig40, S288c-Chr.IX and K7-Chr.IX; B: Dot Matrix Comparison maps of the DNA sequences between *BIO1*-MT1/*BIO6*-MT1 and *BIO1*-Ref/*BIO6*-Ref; C: Dot Matrix Comparison maps of the protein sequences between *BIO1*-MT1/*BIO6*-MT1 and *BIO1*-Ref/*BIO6*-Ref. **Figure S3.** A: Colinearity analysis of Chr. I. The same Locally Collinear Block is represented by same color and vertical lines represent the same LCB; The height of internal vertical lines in LCB on behalf of the level of sequence consistency; Blank in one strain stands for the region does not exist in the other. B: PCR profiles of the 25Kb fragment specific to S288c which was divided into 4 small segments (S1, S2, S3, S4). In addition, 26S rDNA gene was detected as the positive control. M: DNA maker of 5000 bp. (DOC 849 kb)
