# Supplementary material for: Genomic and transcriptomic analyses of the Chinese Maotai-flavored liquor yeast MT1 revealed its unique multi-carbon co-utilization
Source: BMC Genomics. 2015 Dec 15;16:1064. doi: 10.1186/s12864-015-2263-0 (PMC4678718; doi:10.1186/s12864-015-2263-0)
Supplement: Additional file 1: Table S1. — Statistics of the SNPs and Indels among the MT1 Chromosomes. Table S2. Annotation of SNPs. Table S3. Annotation of Indels. Table S4. Copy Number Variations (CNVs) between MT1 and S288c. Table S5. Functions of the missed genes. Figure S1. Statistical analysis on the number of contigs with identity <95 % in the amino acid levels. Figure S2. Analysis of two BIO genes present in the LI genome, but not in the S288c genome. A: Schematic organization of two relevant segments from MT1-contig40, S288c-Chr.IX and K7-Chr.IX; B: Dot Matrix Comparison maps of the DNA sequences between BIO1-MT1/BIO6-MT1 and BIO1-Ref/BIO6-Ref; C: Dot Matrix Comparison maps of the protein sequences between BIO1-MT1/BIO6-MT1 and BIO1-Ref/BIO6-Ref. Figure S3. A: Colinearity analysis of Chr. I. The same Locally Collinear Block is represented by same color and vertical lines represent the same LCB; The height of internal vertical lines in LCB on behalf of the level of sequence consistency; Blank in one strain stands for the region does not exist in the other. B: PCR profiles of the 25Kb fragment specific to S288c which was divided into 4 small segments (S1, S2, S3, S4). In addition, 26S rDNA gene was detected as the positive control. M: DNA maker of 5000 bp. (DOC 849 kb) [file 12864_2015_2263_MOESM1_ESM.doc]

**Additional Tables**

**Table S1**. Statistics of the SNPs and Indels among the MT1 Chromosomes

| Chromosome | Chr. Covered (%) | Homo. SNP Number | Heter. SNP Number | Total SNP Number | Homo. INDEL Number | Heter. INDEL Number | Total INDEL Number |
| --- | --- | --- | --- | --- | --- | --- | --- |
| I | 84.624 | 740 | 292 | 1032 | 88 | 72 | 160 |
| II | 99.205 | 2489 | 538 | 3027 | 228 | 95 | 323 |
| III | 99.804 | 1404 | 329 | 1733 | 156 | 91 | 247 |
| IV | 98.914 | 6323 | 1858 | 8181 | 547 | 265 | 812 |
| V | 99.603 | 2331 | 674 | 3005 | 225 | 109 | 334 |
| VI | 91.477 | 894 | 92 | 986 | 105 | 25 | 130 |
| VII | 99.667 | 4413 | 1575 | 5988 | 381 | 218 | 599 |
| VIII | 94.732 | 2043 | 1063 | 3106 | 195 | 110 | 305 |
| IX | 99.175 | 1354 | 878 | 2232 | 158 | 105 | 263 |
| X | 98.974 | 2196 | 1170 | 3366 | 177 | 129 | 306 |
| XI | 99.844 | 2263 | 856 | 3119 | 210 | 137 | 347 |
| XII | 97.568 | 3537 | 629 | 4166 | 328 | 99 | 427 |
| XIII | 99.481 | 3165 | 1241 | 4406 | 272 | 137 | 409 |
| XIV | 98.12 | 3388 | 780 | 4168 | 299 | 120 | 419 |
| XV | 98.568 | 4165 | 765 | 4930 | 379 | 103 | 482 |
| XVI | 98.762 | 4094 | 1373 | 5467 | 371 | 119 | 490 |
| Mito | 90.615 | 48 | 0 | 48 | 338 | 83 | 421 |
| Summary | 98.263 | 44847 | 14113 | 58960 | 4457 | 2017 | 6474 |

**Table S2**. Annotation of SNPs

| Type | Number |
| --- | --- |
| 3_prime_UTR_variant | 64 |
| downstream_gene_variant | 175163 |
| initiator_codon_variant | 24 |
| intron_variant | 415 |
| intron_variant,nc_transcript_variant | 13 |
| missense_variant | 13780 |
| non_coding_exon_variant,nc_transcript_variant | 214 |
| splice_acceptor_variant,nc_transcript_variant | 1 |
| splice_donor_variant | 1 |
| splice_donor_variant,nc_transcript_variant | 2 |
| splice_region_variant,intron_variant | 12 |
| splice_region_variant,intron_variant,nc_transcript_variant | 2 |
| stop_gained | 105 |
| stop_lost | 1 |
| synonymous_variant | 25724 |
| upstream_gene_variant | 178294 |

**Table S3**. Annotation of Indels

| Type | Number |
| --- | --- |
| 3_prime_UTR_variant,feature_elongation | 10 |
| 3_prime_UTR_variant,feature_truncation | 9 |
| coding_sequence_variant,3_prime_UTR_variant,feature_truncation | 4 |
| coding_sequence_variant,5_prime_UTR_variant | 2 |
| coding_sequence_variant,5_prime_UTR_variant,feature_truncation | 8 |
| downstream_gene_variant | 21971 |
| downstream_gene_variant,feature_elongation | 10 |
| frameshift_variant | 9 |
| frameshift_variant,feature_elongation | 251 |
| frameshift_variant,feature_truncation | 262 |
| inframe_deletion | 227 |
| inframe_insertion | 221 |
| initiator_codon_variant,inframe_insertion | 1 |
| intron_variant | 3 |
| intron_variant,feature_elongation | 78 |
| intron_variant,feature_truncation | 85 |
| intron_variant,nc_transcript_variant,feature_elongation | 3 |
| intron_variant,nc_transcript_variant,feature_truncation | 1 |
| missense_variant | 7 |
| missense_variant,feature_elongation | 15 |
| missense_variant,feature_truncation | 25 |
| non_coding_exon_variant,nc_transcript_variant | 1 |
| non_coding_exon_variant,nc_transcript_variant,feature_elongation | 16 |
| non_coding_exon_variant,nc_transcript_variant,feature_truncation | 28 |
| splice_donor_variant,coding_sequence_variant,intron_variant,feature_truncation | 1 |
| stop_gained,feature_elongation | 1 |
| stop_gained,inframe_insertion | 3 |
| upstream_gene_variant | 23399 |
| upstream_gene_variant,feature_elongation | 3 |

**Table S4**. Copy Number Variations (CNVs) between MT1 and S288c.

| Variation Type | Region | Size |
| --- | --- | --- |
| deletion | Mito:14001-18900 | 4900 |
| duplication | Mito:18901-21000 | 2100 |
| deletion | Mito:21101-23600 | 2500 |
| deletion | Mito:37001-39100 | 2100 |
| duplication | Mito:39101-50200 | 11100 |
| duplication | Mito:50501-60700 | 10200 |
| deletion | Mito:60801-61800 | 1000 |
| deletion | Mito:81901-82300 | 400 |
| duplication | chrI:1-12200 | 12200 |
| deletion | chrI:12201-30200 | 18000 |
| deletion | chrI:68001-72000 | 4000 |
| deletion | chrI:160201-166200 | 6000 |
| deletion | chrI:181401-184900 | 3500 |
| deletion | chrI:197501-230300 | 32800 |
| deletion | chrVI:2501-4700 | 2200 |
| deletion | chrVI:7101-8300 | 1200 |
| deletion | chrVI:8601-11100 | 2500 |
| deletion | chrVI:13701-34400 | 20700 |
| duplication | chrVI:139001-145500 | 6500 |
| deletion | chrVI:267201-270200 | 3000 |
| duplication | III:1401-4000 | 2600 |
| deletion | III:56701-57500 | 800 |
| deletion | III:82301-83100 | 800 |
| deletion | III:84001-84900 | 900 |
| duplication | III:89201-90600 | 1400 |
| deletion | III:124101-124500 | 400 |
| deletion | III:168401-169600 | 1200 |
| deletion | III:303801-316700 | 12900 |
| duplication | chrIX:1-6900 | 6900 |
| deletion | chrIX:8501-16900 | 8400 |
| deletion | chrIX:25501-25800 | 300 |
| deletion | chrIX:206101-208900 | 2800 |
| deletion | chrIX:209301-210300 | 1000 |
| deletion | chrIX:300501-301400 | 900 |
| deletion | chrIX:433601-439900 | 6300 |
| deletion | VIII:1-700 | 700 |
| deletion | VIII:2201-8400 | 6200 |
| deletion | VIII:12201-12700 | 500 |
| deletion | VIII:85201-95000 | 9800 |
| deletion | VIII:116401-116800 | 400 |
| deletion | VIII:146301-146700 | 400 |
| deletion | VIII:212401-216000 | 3600 |
| deletion | VIII:293001-293600 | 600 |
| deletion | VIII:522501-547100 | 24600 |
| deletion | VIII:548601-553700 | 5100 |
| deletion | VIII:556001-560500 | 4500 |
| deletion | chrV:249901-250300 | 400 |
| deletion | chrV:354301-354800 | 500 |
| deletion | chrV:435901-436300 | 400 |
| deletion | chrV:443301-450000 | 6700 |
| deletion | chrV:487801-488200 | 400 |
| deletion | chrV:493801-495900 | 2100 |
| deletion | chrV:571401-576900 | 5500 |
| deletion | chrXI:101-1300 | 1200 |
| deletion | chrXI:1701-2800 | 1100 |
| deletion | chrXI:15901-16300 | 400 |
| deletion | chrXI:258501-261400 | 2900 |
| deletion | chrXI:660401-662200 | 1800 |
| duplication | chrX:1-6900 | 6900 |
| deletion | chrX:8301-17500 | 9200 |
| deletion | chrX:197801-204200 | 6400 |
| deletion | chrX:354801-355200 | 400 |
| deletion | chrX:472501-473300 | 800 |
| deletion | chrX:473401-479500 | 6100 |
| deletion | chrX:480401-483500 | 3100 |
| deletion | chrX:726801-743400 | 16600 |
| deletion | chrX:743601-745000 | 1400 |
| deletion | XIV:1-9000 | 9000 |
| deletion | XIV:96601-102300 | 5700 |
| deletion | XIV:519001-522600 | 3600 |
| deletion | XIV:547001-547900 | 900 |
| deletion | XIV:567101-568300 | 1200 |
| deletion | XIV:571501-573600 | 2100 |
| duplication | XIV:598501-600700 | 2200 |
| deletion | XIV:604101-606600 | 2500 |
| deletion | XIV:750601-751400 | 800 |
| duplication | XIV:756001-758300 | 2300 |
| deletion | XIV:765401-779400 | 14000 |
| deletion | chrII:1901-7600 | 5700 |
| deletion | chrII:8901-9500 | 600 |
| deletion | chrII:29601-36000 | 6400 |
| deletion | chrII:165601-166100 | 500 |
| duplication | chrII:221401-226900 | 5500 |
| deletion | chrII:428101-429900 | 1800 |
| deletion | chrII:643801-645000 | 1200 |
| duplication | chrII:780001-783200 | 3200 |
| duplication | chrII:801401-805000 | 3600 |
| deletion | chrII:807101-813200 | 6100 |
| deletion | XIII:1-8400 | 8400 |
| deletion | XIII:157701-159300 | 1600 |
| deletion | XIII:186501-188000 | 1500 |
| deletion | XIII:188601-189900 | 1300 |
| deletion | XIII:196301-200800 | 4500 |
| deletion | XIII:372701-376300 | 3600 |
| deletion | XIII:625301-627000 | 1700 |
| deletion | XIII:808501-808900 | 400 |
| duplication | XIII:906901-909700 | 2800 |
| deletion | XIII:917201-918000 | 800 |
| deletion | XVI:1-20300 | 20300 |
| deletion | XVI:56401-63000 | 6600 |
| deletion | XVI:211601-211900 | 300 |
| duplication | XVI:406601-411200 | 4600 |
| deletion | XVI:436901-443200 | 6300 |
| deletion | XVI:769401-769700 | 300 |
| deletion | XVI:776901-781600 | 4700 |
| deletion | XVI:804601-807900 | 3300 |
| deletion | XVI:809101-810600 | 1500 |
| deletion | XVI:838101-839300 | 1200 |
| deletion | XVI:846801-848300 | 1500 |
| deletion | XVI:848901-857000 | 8100 |
| duplication | XVI:880701-882300 | 1600 |
| duplication | XVI:931701-933600 | 1900 |
| deletion | XVI:942301-948100 | 5800 |
| deletion | XII:1-13900 | 13900 |
| deletion | XII:216501-218500 | 2000 |
| deletion | XII:374001-374300 | 300 |
| duplication | XII:451701-468800 | 17100 |
| deletion | XII:468801-490000 | 21200 |
| deletion | XII:593101-599100 | 6000 |
| deletion | XII:650801-657000 | 6200 |
| deletion | XII:945801-946900 | 1100 |
| deletion | XII:976001-980000 | 4000 |
| deletion | XII:1065301-1066000 | 700 |
| deletion | XII:1067801-1078200 | 10400 |
| deletion | VII:7001-9200 | 2200 |
| deletion | VII:75701-76000 | 300 |
| deletion | VII:89401-90000 | 600 |
| deletion | VII:319401-319800 | 400 |
| deletion | VII:323301-325400 | 2100 |
| deletion | VII:401601-401900 | 300 |
| deletion | VII:402301-405300 | 3000 |
| deletion | VII:482301-486100 | 3800 |
| deletion | VII:535501-540000 | 4500 |
| deletion | VII:561901-566300 | 4400 |
| deletion | VII:567401-567800 | 400 |
| duplication | VII:568901-571000 | 2100 |
| deletion | VII:573001-574800 | 1800 |
| deletion | VII:707701-708000 | 300 |
| duplication | VII:708001-712200 | 4200 |
| deletion | VII:735901-736400 | 500 |
| duplication | VII:812501-821200 | 8700 |
| deletion | VII:930801-932100 | 1300 |
| duplication | VII:1072601-1081800 | 9200 |
| deletion | VII:1082801-1091000 | 8200 |
| deletion | chrXV:1-12000 | 12000 |
| duplication | chrXV:12001-17700 | 5700 |
| deletion | chrXV:17801-19200 | 1400 |
| duplication | chrXV:19201-21300 | 2100 |
| duplication | chrXV:28801-31200 | 2400 |
| deletion | chrXV:42901-43800 | 900 |
| deletion | chrXV:117701-123600 | 5900 |
| deletion | chrXV:497101-497400 | 300 |
| duplication | chrXV:598701-600700 | 2000 |
| deletion | chrXV:664801-665100 | 300 |
| deletion | chrXV:703401-710400 | 7000 |
| duplication | chrXV:755801-758900 | 3100 |
| deletion | chrXV:970301-976300 | 6000 |
| deletion | chrXV:1069901-1071700 | 1800 |
| duplication | chrXV:1075001-1077300 | 2300 |
| deletion | chrXV:1077301-1091300 | 14000 |
| deletion | chrIV:101-2200 | 2100 |
| deletion | chrIV:5601-16900 | 11300 |
| duplication | chrIV:348601-351100 | 2500 |
| deletion | chrIV:434201-434900 | 700 |
| deletion | chrIV:437101-437600 | 500 |
| deletion | chrIV:514201-516700 | 2500 |
| deletion | chrIV:518101-521400 | 3300 |
| deletion | chrIV:522601-538300 | 15700 |
| deletion | chrIV:645201-649900 | 4700 |
| deletion | chrIV:668001-668500 | 500 |
| deletion | chrIV:802901-805500 | 2600 |
| deletion | chrIV:871801-880600 | 8800 |
| deletion | chrIV:980801-990400 | 9600 |
| deletion | chrIV:991201-992700 | 1500 |
| deletion | chrIV:1023101-1023500 | 400 |
| deletion | chrIV:1098001-1099600 | 1600 |
| deletion | chrIV:1100201-1101700 | 1500 |
| deletion | chrIV:1154901-1155600 | 700 |
| deletion | chrIV:1155801-1160100 | 4300 |
| deletion | chrIV:1160301-1163100 | 2800 |
| deletion | chrIV:1206701-1212700 | 6000 |
| deletion | chrIV:1352601-1353000 | 400 |
| deletion | chrIV:1520501-1532000 | 11500 |

**Table S5**. Functions of the missed genes

| Name | SGD ID | Description |
| --- | --- | --- |
| *CTR2* | S000001218 | Putative low-affinity copper transporter of the vacuolar membrane; mutation confers resistance to toxic copper concentrations, while overexpression confers resistance to copper starvation; regulated by nonsense-mediated mRNA decay pathway |
| *CTR9* | S000005505 | Component of the Paf1p complex involved in transcription elongation; binds to and modulates the activity of RNA polymerases I and II; required for expression of a subset of genes, including cyclin genes; involved in SER3 repression by helping to maintain SRG1 transcription-dependent nucleosome occupancy |
| *AIM1* | S000000044 | Protein involved in mitochondrial function or organization; null mutant displays elevated frequency of mitochondrial genome loss |
| *ECM1* | S000000055 | Pre-ribosomal factor involved in 60S ribosomal protein subunit export; associates with the pre-60S particle; shuttles between the nucleus and cytoplasm |
| *MIC23* | S000000203 | Mitochondrial intermembrane space protein of unknown function; imported via the MIA import machinery; contains an unusual twin cysteine motif |
| *MIC17* | S000004604 | Mitochondrial intermembrane space protein; required for normal oxygen consumption; contains twin cysteine-x9-cysteine motifs; protein abundance increases in response to DNA replication stress |
| *RRN3* | S000001608 | Protein required for transcription of rDNA by RNA polymerase I; transcription factor independent of DNA template; involved in recruitment of RNA polymerase I to rDNA; structure reveals unique HEAT repeat fold and a surface serine patch; phosphorylation of serine patch impairs cell growth and reduces RNA polymerase I binding in vitro and RNA polymerase I recruitment to the rDNA gene in vivo |
| *RRN6* | S000000110 | Component of the core factor (CF) rDNA transcription factor complex; CF is required for transcription of 35S rRNA genes by RNA polymerase I and is composed of Rrn6p, Rrn7p, and Rrn11p |
| *TIM12* | S000000295 | Essential protein of the inner mitochondrial membrane; peripherally localized; component of the TIM22 complex, which is a twin-pore translocase that mediates insertion of numerous multispanning inner membrane proteins |
| *TIM8* | S000007348 | Mitochondrial intermembrane space protein; forms a complex with Tim13p that delivers a subset of hydrophobic proteins to the TIM22 complex for inner membrane insertion; homolog of human TIMM8A, implicated in Mohr-Tranebjaerg syndrome |
| *VMA2* | S000000331 | Subunit B of V1 peripheral membrane domain of vacuolar H+-ATPase; an electrogenic proton pump found throughout the endomembrane system; contains nucleotide binding sites; also detected in the cytoplasm; protein abundance increases in response to DNA replication stress |
| *VMA9* | S000028508 | Vacuolar H+ ATPase subunit e of the V-ATPase V0 subcomplex; essential for vacuolar acidification; interacts with the V-ATPase assembly factor Vma21p in the ER; involved in V0 biogenesis |
| *VMA4* | S000005859 | Subunit E of the V1 domain of the vacuolar H+-ATPase (V-ATPase); V-ATPase is an electrogenic proton pump found throughout the endomembrane system; V1 domain has eight subunits; required for the V1 domain to assemble onto the vacuolar membrane; protein abundance increases in response to DNA replication stress |
| *ERV1* | S000003261 | Flavin-linked sulfhydryl oxidase of the mitochondrial IMS; N-terminus is an intrinsically disordered domain that in the cytosol helps target Erv1p to mitochondria, and in the intermembrane space oxidizes Mia40p as part of a disulfide relay system that promotes intermembrane space retention of imported proteins; functional ortholog of human GFER (ALR) |
| *ERV15* | S000000414 | Protein involved in export of proteins from the endoplasmic reticulum |
| *DAD3* | S000007595 | Essential subunit of the Dam1 complex (aka DASH complex); complex couples kinetochores to the force produced by MT depolymerization thereby aiding in chromosome segregation; is transferred to the kinetochore prior to mitosis |
| *DAD2* | S000001791 | Essential subunit of the Dam1 complex (aka DASH complex); complex couples kinetochores to the force produced by MT depolymerization thereby aiding in chromosome segregation; is transferred to the kinetochore prior to mitosis |
| *DAD4* | S000007604 | Essential subunit of the Dam1 complex (aka DASH complex); complex couples kinetochores to the force produced by MT depolymerization thereby aiding in chromosome segregation; is transferred to the kinetochore prior to mitosis |
| *MTC4* | S000000459 | Protein of unknown function; required for normal growth rate at 15 degrees C; green fluorescent protein (GFP)-fusion protein localizes to the cytoplasm in a punctate pattern |
| *PEX34* | S000000561 | Protein that regulates peroxisome populations; peroxisomal integral membrane protein; interacts with Pex11p, Pex25p, and Pex27p to control both constitutive peroxisome division and peroxisome morphology and abundance during peroxisome proliferation |
| *PEX7* | S000002549 | Peroxisomal signal receptor for peroxisomal matrix proteins; recognizes the N-terminal nonapeptide signal (PTS2); WD repeat protein; defects in human homolog cause lethal rhizomelic chondrodysplasia punctata (RCDP) |
| *TAF2* | S000000638 | TFIID subunit (150 kDa); involved in RNA polymerase II transcription initiation |
| *TAF11* | S000004477 | TFIID subunit (40 kDa); involved in RNA polymerase II transcription initiation |
| *TAF12* | S000002552 | Subunit (61/68 kDa) of TFIID and SAGA complexes; involved in RNA polymerase II transcription initiation and in chromatin modification, similar to histone H2A |
| *PAU24* | S000000505 | Cell wall mannoprotein; has similarity to Tir1p, Tir2p, Tir3p, and Tir4p; member of the seripauperin multigene family encoded mainly in subtelomeric regions; expressed under anaerobic conditions, completely repressed during aerobic growth |
| *PAU1* | S000003759 | Member of the seripauperin multigene family; encoded mainly in subtelomeric regions; active during alcoholic fermentation; regulated by anaerobiosis, negatively regulated by oxygen; repressed by heme |
| *PAU6* | S000005359 | Member of the seripauperin multigene family; encoded mainly in subtelomeric regions; active during alcoholic fermentation; regulated by anaerobiosis; negatively regulated by oxygen; repressed by heme |
| *PAU7* | S000000073 | Member of the seripauperin multigene family; active during alcoholic fermentation, regulated by anaerobiosis, inhibited by oxygen, repressed by heme |
| *PAU3* | S000000701 | Member of the seripauperin multigene family; encoded mainly in subtelomeric region; active during alcoholic fermentation; regulated by anaerobiosis; negatively regulated by oxygen; repressed by heme |
| *PAU23* | S000004027 | Cell wall mannoprotein; has similarity to Tir1p, Tir2p, Tir3p, and Tir4p; member of the seripauperin multigene family encoded mainly in subtelomeric regions; expressed under anaerobic conditions, completely repressed during aerobic growth |
| *MAK3* | S000006255 | Catalytic subunit of the NatC type N-terminal acetyltransferase; involved in subcellular targeting of select N-terminally acetylated substrates to the Golgi apparatus (Arl3p and Grh1p) and the inner nuclear membrane (Trm1p); required for maintenance of dsRNA virus |
| *MAK21* | S000002467 | Constituent of 66S pre-ribosomal particles; required for large (60S) ribosomal subunit biogenesis; acts as part of a Mak21p-Noc2p-Rrp5p module that associates with nascent pre-rRNA during transcription and has a role in bigenesis of the large ribosomal subunit; involved in nuclear export of pre-ribosomes; required for maintenance of dsRNA virus |
| *MAK31* | S000000614 | Non-catalytic subunit of N-terminal acetyltransferase of the NatC type; required for replication of dsRNA virus |
| *RDS3* | S000006298 | Component of the SF3b subcomplex of the U2 snRNP; zinc cluster protein involved in pre-mRNA splicing and cycloheximide resistance |
| *TIR1* | S000000813 | Cell wall mannoprotein; expression is downregulated at acidic pH and induced by cold shock and anaerobiosis; abundance is increased in cells cultured without shaking; member of the Srp1p/Tip1p family of serine-alanine-rich proteins |
| *TIR4* | S000005535 | Cell wall mannoprotein; expressed under anaerobic conditions and required for anaerobic growth; transcription is also induced by cold shock; member of the Srp1p/Tip1p family of serine-alanine-rich proteins |
| *LSM4* | S000000914 | Lsm (Like Sm) protein; part of heteroheptameric complexes (Lsm2p-7p and either Lsm1p or 8p): cytoplasmic Lsm1p complex involved in mRNA decay; nuclear Lsm8p complex part of U6 snRNP and possibly involved in processing tRNA, snoRNA, and rRNA; forms cytoplasmic foci upon DNA replication stress |
| *LSM8* | S000003783 | Lsm (Like Sm) protein; forms heteroheptameric complex (with Lsm2p, Lsm3p, Lsm4p, Lsm5p, Lsm6p, and Lsm7p) that is part of spliceosomal U6 snRNP and is also implicated in processing of pre-tRNA, pre-snoRNA, and pre-rRNA |
| *LSM6* | S000002786 | Lsm (Like Sm) protein; part of heteroheptameric complexes (Lsm2p-7p and either Lsm1p or 8p): cytoplasmic Lsm1p complex involved in mRNA decay; nuclear Lsm8p complex part of U6 snRNP and possibly involved in processing tRNA, snoRNA, and rRNA |
| *RAD3* | S000000973 | 5' to 3' DNA helicase; involved in nucleotide excision repair and transcription; subunit of RNA polII initiation factor TFIIH and of Nucleotide Excision Repair Factor 3 (NEF3); homolog of human XPD protein; mutant has aneuploidy tolerance; protein abundance increases in response to DNA replication stress |
| *RAD10* | S000004560 | Single-stranded DNA endonuclease (with Rad1p); cleaves single-stranded DNA during nucleotide excision repair and double-strand break repair; subunit of Nucleotide Excision Repair Factor 1 (NEF1); homolog of human ERCC1 protein |
| *RPB9* | S000003038 | RNA polymerase II subunit B12.6; contacts DNA; mutations affect transcription start site selection and fidelity of transcription |
| *RPB7* | S000002812 | RNA polymerase II subunit B16; forms dissociable heterodimer with Rpb4p; Rpb4/7 dissociates from RNAPII as Ser2 CTD phosphorylation increases; Rpb4/7 regulates cellular lifespan via mRNA decay process; involved in recruitment of 3'-end processing factors to transcribing RNA polymerase II complex, export of mRNA to cytoplasm under stress conditions; also involved in translation initiation |
| *AGA2* | S000003000 | Adhesion subunit of a-agglutinin of a-cells; C-terminal sequence acts as a ligand for alpha-agglutinin (Sag1p) during agglutination, modified with O-linked oligomannosyl chains, linked to anchorage subunit Aga1p via two disulfide bonds |
| *AGA1* | S000005327 | Anchorage subunit of a-agglutinin of a-cells; highly O-glycosylated protein with N-terminal secretion signal and C-terminal signal for addition of GPI anchor to cell wall, linked to adhesion subunit Aga2p via two disulfide bonds |
| *PRP18* | S000003238 | Splicing factor and component of snRNP U5; factor involved in the positioning of the 3' splice site during the second catalytic step of splicing; interacts with Slu7p |
| *PRP3* | S000002881 | Splicing factor; component of the U4/U6-U5 snRNP complex |
| *ERV15* | S000000414 | Protein involved in export of proteins from the endoplasmic reticulum |
| *ERV1* | S000003261 | Flavin-linked sulfhydryl oxidase of the mitochondrial IMS; N-terminus is an intrinsically disordered domain that in the cytosol helps target Erv1p to mitochondria, and in the intermembrane space oxidizes Mia40p as part of a disulfide relay system that promotes intermembrane space retention of imported proteins; functional ortholog of human GFER |
| *NOP7* | S000003335 | Component of several different pre-ribosomal particles; forms a complex with Ytm1p and Erb1p that is required for maturation of the large ribosomal subunit; required for exit from G0 and the initiation of cell proliferation |
| *NOP2* | S000005005 | rRNA m5C methyltransferase; methylates cytosine at position 2870 of 25S rRNA while Rcm1p methylates cytosine at position 2278; contains seven beta-strand methyltransferase motif; essential for processing and maturation of 27S pre-rRNA and large ribosomal subunit biogenesis; localized to the nucleolus; constituent of 66S pre-ribosomal particles |
| *RTT102* | S000003507 | Component of both the SWI/SNF and RSC chromatin remodeling complexes; suggested role in chromosome maintenance; possible weak regulator of Ty1 transposition; protein abundance increases in response to DNA replication stress |
| *RTT10* | S000006104 | WD40 domain-containing protein involved in endosomal recycling; forms a complex with Rrt2p that functions in the retromer-mediated pathway for recycling internalized cell-surface proteins; interacts with Trm7p for 2'-O-methylation of N34 of substrate tRNAs; has a role in regulation of Ty1 transposition |
| *RPA34* | S000003684 | RNA polymerase I subunit A34.5; essential for nucleolar assembly and for high polymerase loading rate |
| *RPA12* | S000003824 | RNA polymerase I subunit A12.2; contains two zinc binding domains, and the N terminal domain is responsible for anchoring to the RNA pol I complex |
| *SET2* | S000003704 | Histone methyltransferase with a role in transcriptional elongation; methylates H3 lysine 36 (H3K36), which suppresses incorporation of acetylated histones and signals for the deacetylation of these histones within transcribed genes; associates with the C-terminal domain(CTD) of Rpo21p; H3K36me3 (trimethylation) requires Spt6p, proline 38 on H3, CTD of Rpo21p, Ctk1p, and C-terminal SRI domain of Ste2p; relocalizes to the cytosol in response to hypoxia |
| *COX16* | S000003540 | Mitochondrial inner membrane protein; required for assembly of cytochrome c oxidase |
| *COX17* | S000003932 | Copper metallochaperone that transfers copper to Sco1p and Cox11p; eventual delivery to cytochrome c oxidase; contains twin cysteine-x9-cysteine motifs |
| *SRP21* | S000001605 | Subunit of the signal recognition particle (SRP); SRP functions in protein targeting to the endoplasmic reticulum membrane; not found in mammalian SRP; forms a pre-SRP structure in the nucleolus that is translocated to the cytoplasm |
| *SRP40* | S000001800 | Nucleolar serine-rich protein; role in preribosome assembly or transport; may function as a chaperone of small nucleolar ribonucleoprotein particles (snoRNPs); immunologically and structurally to rat Nopp140 |
| *SRP54* | S000006292 | Signal recognition particle (SRP) subunit (homolog of mammalian SRP54); contains the signal sequence-binding activity of SRP, interacts with the SRP RNA, and mediates binding of SRP to signal receptor; contains GTPase domain |
| *CWP2* | S000001956 | Covalently linked cell wall mannoprotein; major constituent of the cell wall; plays a role in stabilizing the cell wall; involved in low pH resistance; precursor is GPI-anchored |
| *CWP1* | S000001579 | Cell wall mannoprotein that localizes to birth scars of daughter cells; linked to a beta-1,3- and beta-1,6-glucan heteropolymer through a phosphodiester bond; required for propionic acid resistance |
| *COA2* | S000028527 | Cytochrome oxidase assembly factor; null mutation results in respiratory deficiency with specific loss of cytochrome oxidase activity; functions downstream of assembly factors Mss51p and Coa1p and interacts with assembly factor Shy1p |
| *COA4* | S000004208 | win Cx(9)C protein involved in cytochrome c oxidase organization; organization includes assembly or stability; localizes to the mitochondrial intermembrane space via the Mia40p-Erv1p system; interacts genetically with CYC1 and with cytochrome c oxidase assembly factors |
| *VPS38* | S000004352 | Part of a Vps34p phosphatidylinositol 3-kinase complex; functions in carboxypeptidase Y (CPY) sorting; binds Vps30p and Vps34p to promote production of phosphatidylinositol 3-phosphate (PtdIns3P) which stimulates kinase activity; required for overflow degradation of misfolded proteins when ERAD is saturated |
| *VPS60* | S000002894 | Protein involved in late endosome to vacuole transport; cytoplasmic and vacuolar membrane protein; required for normal filament maturation during pseudohyphal growth; may function in targeting cargo proteins for degradation |
| *GPI15* | S000004983 | Protein involved in the synthesis of GlcNAc-PI; GlcNAc-PI is the first intermediate in the synthesis of glycosylphosphatidylinositol (GPI) anchors |
| *NPR3* | S000001015 | Subunit of SEA (Seh1-associated), Npr2/3, and Iml1p complexes; Npr2/3 complex mediates downregulation of TORC1 activity upon amino acid limitation; SEA complex is a coatomer-related complex that associates dynamically with the vacuole; Iml1p complex (Iml1p-Npr2p-Npr3p) is required for non-nitrogen-starvation (NNS)-induced autophagy; required for Npr2p phosphorylation and Iml1p-Npr2p interaction; null mutant shows delayed meiotic DNA replication and double-strand break repair |
| *SHU1* | S000000998 | Component of Shu complex (aka PCSS complex); Shu complex also includes Psy3, Csm2, Shu2, and promotes error-free DNA repair, mediates inhibition of Srs2p function; essential for promoting the establishment of homolog bias during meiotic homologous recombination; promotes both crossover (CO) and non-crossover (NCO) pathways of meiotic recombination and formation of Rad51p filaments |
| *ARD1* | S000001055 | Subunit of protein N-terminal acetyltransferase NatA; NatA is comprised of Nat1p, Ard1p, and Nat5p; acetylates many proteins and thus affects telomeric silencing, cell cycle, heat-shock resistance, mating, and sporulation; human Ard1p levels are elevated in cancer cells; protein abundance increases in response to DNA replication stress |
| *ECM1* | S000000055 | Pre-ribosomal factor involved in 60S ribosomal protein subunit export; associates with the pre-60S particle; shuttles between the nucleus and cytoplasm |
| *NMD2* | S000001119 | Protein involved in the nonsense-mediated mRNA decay (NMD) pathway; interacts with Nam7p and Upf3p; involved in telomere maintenance |
| *IRE1* | S000001121 | Serine-threonine kinase and endoribonuclease; transmembrane protein that mediates the unfolded protein response (UPR) by regulating Hac1p synthesis through HAC1 mRNA splicing; role in homeostatic adaptation to ER stress; Kar2p binds inactive Ire1p and releases from it upon ER stress |
| *LRP1* | S000001123 | Nuclear exosome-associated nucleic acid binding protein; involved in RNA processing, surveillance, degradation, tethering, and export; forms a stable heterodimer with Rrp6p and regulates its exonucleolytic activity; rapidly degraded by the proteasome in the absence of Rrp6p; homolog of mammalian nuclear matrix protein C1D involved in regulation of DNA repair and recombination |
| *KSP1* | S000001124 | Serine/threonine protein kinase; associates with TORC1 and likely involved in TOR signaling cascades; negative regulator of autophagy; nuclear translocation required for haploid filamentous growth; regulates filamentous growth induced nuclear translocation of Bcy1p, Fus3p, and Sks1p; overproduction causes allele-specific suppression of prp20-10; protein abundance increases in response to DNA replication stress |
| *GAR1* | S000001131 | Protein component of the H/ACA snoRNP pseudouridylase complex; involved in the modification and cleavage of the 18S pre-rRNA |
| *YNG2* | S000001132 | Subunit of NuA4, an essential histone acetyltransferase complex; positions Piccolo NuA4 for efficient acetylation of histone H4 or histone H2A; relocalizes to the cytosol in response to hypoxia; similar to human tumor suppressor ING1 and its isoforms ING4 and ING5 |
| *HSP33* | S000005918 | Possible chaperone and cysteine protease; required for transcriptional reprogramming during the diauxic shift and for survival in stationary phase; similar to E. coli Hsp31 and S. cerevisiae Hsp31p, Hsp32p, and Sno4p; member of the DJ-1/ThiJ/PfpI superfamily, which includes human DJ-1 involved in Parkinson's disease and cancer |
| *HSP32* | S000006201 | Possible chaperone and cysteine protease; required for transcriptional reprogramming during the diauxic shift and for survival in stationary phase |
| *HSP10* | S000005546 | Mitochondrial matrix co-chaperonin; inhibits the ATPase activity of Hsp60p, a mitochondrial chaperonin; involved in protein folding and sorting in the mitochondria |
| *BIG1* | S000001143 | Integral membrane protein of the endoplasmic reticulum; required for normal content of cell wall beta-1,6-glucan |
| *DSE2* | S000001186 | Daughter cell-specific secreted protein with similarity to glucanases; degrades cell wall from the daughter side causing daughter to separate from mother; expression is repressed by cAMP |
| *CUP1-2* | S000001097 | Metallothionein; binds copper and mediates resistance to high concentrations of copper and cadmium; locus is variably amplified in different strains, with two copies, CUP1-1 and CUP1-2, in the genomic sequence reference strain S288C |
| *CUP1-1* | S000001095 | Metallothionein; binds copper and mediates resistance to high concentrations of copper and cadmium; locus is variably amplified in different strains, with two copies, CUP1-1 and CUP1-2, in the genomic sequence reference strain S288C |
| *DNA2* | S000001207 | Tripartite DNA replication factor; has single-stranded DNA-dependent ATPase, ATP-dependent nuclease, and helicase activities; tracking protein for flap cleavage during Okazaki fragment maturation; involved in DNA repair and processing of meiotic DNA double strand breaks; required for normal life span; component of telomeric chromatin, with cell-cycle dependent localization; required for telomerase-dependent telomere synthesis; forms nuclear foci upon DNA replication stress |
| *PTH1* | S000001232 | One of two mitochondrially-localized peptidyl-tRNA hydrolases; dispensable for respiratory growth on rich medium, but required for respiratory growth on minimal medium |
| *PMP1* | S000000619 | Regulatory subunit for the plasma membrane H(+)-ATPase Pma1p; small single-membrane span proteolipid; forms unique helix and positively charged cytoplasmic domain that is able to specifically segregate phosphatidylserines |
| *PMP2* | S000002103 | Proteolipid associated with plasma membrane H(+)-ATPase (Pma1p); regulates plasma membrane H(+)-ATPase activity; protein abundance increases in response to DNA replication stress |
| *MNL1* | S000001247 | Alpha-1,2-specific exomannosidase of the endoplasmic reticulum; in complex with Pdi1p, generates a Man7GlcNac2 oligosaccharide signal on glycoproteins destined for ubiquitin-proteasome degradation |
| *MNN11* | S000003719 | Subunit of a Golgi mannosyltransferase complex |
| *SYN8* | S000000012 | Endosomal SNARE related to mammalian syntaxin 8 |
| *MFA1* | S000002869 | Mating pheromone a-factor; made by a cells; interacts with alpha cells to induce cell cycle arrest and other responses leading to mating; biogenesis involves C-terminal modification, N-terminal proteolysis, and export |
| *MFA2* | S000005089 | Mating pheromone a-factor; made by a cells; interacts with alpha cells to induce cell cycle arrest and other responses leading to mating; biogenesis involves C-terminal modification, N-terminal proteolysis, and export |
| *RFA1* | S000000065 | Subunit of heterotrimeric Replication Protein A (RPA); RPA is a highly conserved single-stranded DNA binding protein involved in DNA replication, repair, and recombination; RPA protects against inappropriate telomere recombination, and upon telomere uncapping, prevents cell proliferation by a checkpoint-independent pathway; role in DNA catenation/decatenation pathway of chromosome disentangling; relocalizes to the cytosol in response to hypoxia |
| *KTI11* | S000007587 | Zn-ribbon protein that co-purifies with Dph1 and Dph2; in a complex required for synthesis of diphthamide on translation factor eEF2 and with Elongator subunits Iki3p, Elp2p, and Elp3p; involved in modification of wobble nucleosides in tRNAs; forms a stable heterodimer with Ats1p |
| *TOD6* | S000000150 | PAC motif binding protein involved in rRNA and ribosome biogenesis; subunit of the RPD3L histone deacetylase complex; Myb-like HTH transcription factor; hypophosphorylated by rapamycin treatment in a Sch9p-dependent manner; activated in stochastic pulses of nuclear localization |
| *NCL1* | S000000120 | S-adenosyl-L-methionine-dependent tRNA: m5C-methyltransferase; methylates cytosine to m5C at several positions in tRNAs and intron-containing pre-tRNAs; increases proportion of tRNALeu(CAA) with m5C at wobble position in response to hydrogen peroxide, causing selective translation of mRNA from genes enriched in TTG codon; loss of NCL1 confers hypersensitivity to oxidative stress |
| *RIF1* | S000000479 | rotein that binds to the Rap1p C-terminus; acts synergistically with Rif2p to help control telomere length and establish telomeric silencing; contributes to resection of DNA double strand breaks (DSBs); deletion results in telomere elongation |
| *SWI4* | S000000913 | DNA binding component of the SBF complex (Swi4p-Swi6p); a transcriptional activator that in concert with MBF (Mbp1-Swi6p) regulates late G1-specific transcription of targets including cyclins and genes required for DNA synthesis and repair; Slt2p-independent regulator of cold growth |
| *YPS5* | S000003228 | rotein with similarity to GPI-anchored aspartic proteases; such proteases are Yap1p and Yap3p |
| *BRR6* | S000003216 | Essential nuclear envelope integral membrane protein; required for nuclear envelope morphology, nuclear pore complex localization, nuclear export; exhibits synthetic lethal genetic interactions with genes involved in lipid metabolism |
| *TAD1* | S000003212 | tRNA-specific adenosine deaminase; deaminates adenosine-37 to inosine in tRNA-Ala |
| *GTS1* | S000003149 | Protein involved in Arf3p regulation and in transcription regulation; localizes to the nucleus and to endocytic patches; contains an N-terminal Zn-finger and ArfGAP homology domain, a C-terminal glutamine-rich region, and a UBA (ubiquitin associated) domain; gts1 mutations affect budding, cell size, heat tolerance, sporulation, life span, ultradian rhythms, endocytosis; expression oscillates in a pattern similar to metabolic oscillations |
| *RCK1* | S000003126 | Protein kinase involved in the response to oxidative stress; identified as suppressor of S. pombe cell cycle checkpoint mutations; RCK1 has a paralog, RCK2, that arose from the whole genome duplication |
| *SOH1* | S000003095 | Subunit of the RNA polymerase II mediator complex; associates with core polymerase subunits to form the RNA polymerase II holoenzyme; involved in telomere maintenance; conserved with other metazoan MED31 subunits |
| *NAB2* | S000003090 | Nuclear polyadenylated RNA-binding protein; required for nuclear mRNA export and poly(A) tail length control; binds nuclear pore protein Mlp1p; involved in forming export-competent mRNPs in the nucleus; autoregulates mRNA levels; related to human hnRNPs; nuclear localization sequence binds Kap104p; protein abundance increases in response to DNA replication stress |
| *FMP37* | S000003048 | Highly conserved subunit of the mitochondrial pyruvate carrier; a mitochondrial inner membrane complex comprised of Mpc1p and either Mpc2p or Mpc3p mediates mitochondrial pyruvate uptake; null mutant displays slow growth that is complemented by expression of human or mouse ortholog; mutation in human ortholog is associated with lactic acidosis and hyperpyruvatemia |
| *HSF1* | S000003041 | Trimeric heat shock transcription factor; activates multiple genes in response to highly diverse stresses, including hyperthermia; recognizes variable heat shock elements (HSEs) consisting of inverted NGAAN repeats; monitors translational status of cell at the ribosome through an RQC (Ribosomal Quality Control)-mediated translation-stress signal; involved in diauxic shift; posttranslationally regulated |
| *PRM8* | S000003021 | Pheromone-regulated protein; contains with 2 predicted transmembrane segments and an FF sequence, a motif involved in COPII binding; forms a complex with Prp9p in the ER; member of DUP240 gene family |
| *MST27* | S000003019 | Putative integral membrane protein, involved in vesicle formation; forms complex with Mst28p; member of DUP240 gene family; binds COPI and COPII vesicles |
| *PUF4* | S000002982 | Member of the PUF protein family; PUF family is defined by the presence of Pumilio homology domains that confer RNA binding activity; preferentially binds mRNAs encoding nucleolar ribosomal RNA-processing factors |
| *NAG1* | S000028636 | Protein involved in yeast cell wall biogenesis; localizes to the cell periphery; production of Nag1p is dependent upon the presence of Slt2p and Rlm1p; gene is nested within and antisense to IMO32 |
| *FIG2* | S000000685 | Cell wall adhesin, expressed specifically during mating; may be involved in maintenance of cell wall integrity during mating |
| *HMRA2* | S000000692 | Silenced copy of a2 at HMR; similarity to Alpha2p; required along with a1p for inhibiting expression of the HO endonuclease in a/alpha HO/HO diploid cells with an active mating-type interconversion system |
| *SMD1* | S000003306 | Core Sm protein Sm D1; part of heteroheptameric complex (with Smb1p, Smd2p, Smd3p, Sme1p, Smx3p, and Smx2p) that is part of the spliceosomal U1, U2, U4, and U5 snRNPs; relocalizes to the cytosol in response to hypoxia; homolog of human Sm D1; protein abundance increases in response to DNA replication stress |
| *ESP1* | S000003330 | Separase, a caspase-like cysteine protease; promotes sister chromatid separation by mediating dissociation of the cohesin Scc1p from chromatin; inhibits protein phosphatase 2A-Cdc55p to promote mitotic exit; inhibited by Pds1p; relative distribution to the nucleus increases upon DNA replication stress |
| *NSR1* | S000003391 | Nucleolar protein that binds nuclear localization sequences; required for pre-rRNA processing and ribosome biogenesis |
| *BNS1* | S000003462 | Protein of unknown function; overexpression bypasses need for Spo12p, but not required for meiosis; BNS1 has a paralog, SPO12, that arose from the whole genome duplication |
| *TAO3* | S000001391 | Component of the RAM signaling network; is involved in regulation of Ace2p activity and cellular morphogenesis, interacts with protein kinase Cbk1p and also with Kic1p |
| *SDP1* | S000001375 | Stress-inducible dual-specificity MAP kinase phosphatase; negatively regulates Slt2p MAP kinase by direct dephosphorylation, diffuse localization under normal conditions shifts to punctate localization after heat shock |
| *ARC15* | S000001324 | Subunit of the ARP2/3 complex; ARP2/3 is required for the motility and integrity of cortical actin patches; has mRNA binding activity |
| *ULP2* | S000001293 | Peptidase that deconjugates Smt3/SUMO-1 peptides from proteins; plays a role in chromosome cohesion at centromeric regions and recovery from checkpoint arrest induced by DNA damage or DNA replication defects |
| *EST3* | S000006432 | Component of the telomerase holoenzyme; involved in telomere replication |
| *ASG7* | S000003706 | Protein that regulates signaling from G protein beta subunit Ste4p; contributes to relocalization of Ste4p within the cell; specific to a-cells and induced by alpha-factor |
| *YAK1* | S000003677 | Serine-threonine protein kinase; component of a glucose-sensing system that inhibits growth in response to glucose availability; upon nutrient deprivation Yak1p phosphorylates Pop2p to regulate mRNA deadenylation, the co-repressor Crf1p to inhibit transcription of ribosomal genes, and the stress-responsive transcription factors Hsf1p and Msn2p; nuclear localization negatively regulated by the Ras/PKA signaling pathway in the presence of glucose |
| *PAM16* | S000003640 | Subunit of the import motor (PAM complex); the PAM complex is a component of the Translocase of the Inner Mitochondrial membrane (TIM23 complex); forms a 1:1 subcomplex with Pam18p and inhibits its cochaperone activity; contains a J-like domain |
| *PRY1* | S000003615 | Sterol binding protein involved in the export of acetylated sterols; secreted glycoprotein and member of the CAP protein superfamily (cysteine-rich secretory proteins (CRISP), antigen 5, and pathogenesis related 1 proteins); sterol export function is redundant with that of PRY2; may be involved in detoxification of hydrophobic compounds |
| *ICS3* | S000003613 | Protein with a role in processing of secretory proteins; possible role in vacuolar sorting, null mutants are hypersensitive to sortin2 |
| *LOH1* | S000003575 | Protein involved in outer spore wall assembly; likely involved directly in dityrosine layer assembly; proposed role in maintenance of genome integrity; induced during sporulation; repressed during vegetative growth by Sum1p and Hst1p; sequence similar to adjacent ORF, IRC18/YJL037W, and the double mutant irc18 loh1 exhibits reduced dityrosine fluorescence relative to the single mutants |
| *BBC1* | S000003557 | Protein possibly involved in assembly of actin patches; interacts with an actin assembly factor Las17p and with the SH3 domains of Type I myosins Myo3p and Myo5p; localized predominantly to cortical actin patches |
| *MPS3* | S000003556 | Nuclear envelope protein; required for SPB insertion, SPB duplication, Kar5p localization near the SPB and nuclear fusion; interacts with Mps2p to tether half-bridge to core SPB; N-terminal acetylation by Eco1p regulates its role in nuclear organization; localizes to the SPB half bridge and telomeres during meiosis; required with Ndj1p and Csm4p for meiotic bouquet formation and telomere-led rapid prophase movement |
| *SYS1* | S000003541 | Integral membrane protein of the Golgi; required for targeting of the Arf-like GTPase Arl3p to the Golgi; multicopy suppressor of ypt6 null mutation |
| *AVT1* | S000003761 | Vacuolar transporter; imports large neutral amino acids into the vacuole; member of a family of seven S. cerevisiae genes (AVT1-7) related to vesicular GABA-glycine transporters |
| *MOG1* | S000003835 | Conserved nuclear protein that interacts with GTP-Gsp1p; stimulates nucleotide release from Gsp1p; involved in nuclear protein import; nucleotide release is inhibited by Yrb1p |
| *EMC2* | S000003848 | Member of conserved ER transmembrane complex; required for efficient folding of proteins in the ER; null mutant displays induction of the unfolded protein response |
| *IBA57* | S000003883 | Protein involved in incorporating iron-sulfur clusters into proteins; mitochondrial matrix protein; involved in the incorporation of iron-sulfur clusters into mitochondrial aconitase-type proteins; activates the radical-SAM family members Bio2p and Lip5p; interacts with Ccr4p in the two-hybrid system |
| *CNB1* | S000001673 | Calcineurin B; regulatory subunit of calcineurin, a Ca++/calmodulin-regulated type 2B protein phosphatase which regulates Crz1p (stress-response transcription factor); other calcineurin subunit encoded by CNA1 and/or CMP1; regulates function of Aly1p alpha-arrestin; myristoylation by Nmt1p reduces calcineurin activity in response to submaximal Ca signals, is needed to prevent constitutive phosphatase activity; protein abundance increases in response to DNA replication stress |
| *TPO5* | S000001657 | Protein involved in excretion of putrescine and spermidine; putative polyamine transporter in the Golgi or post-Golgi vesicles |
| *ELF1* | S000001643 | Transcription elongation factor with a conserved zinc finger domain; implicated in the maintenance of proper chromatin structure in actively transcribed regions; deletion inhibits Brome mosaic virus (BMV) gene expression |
| *SHE2* | S000001613 | RNA-binding protein that binds specific mRNAs and interacts with She3p; part of the mRNA localization machinery that restricts accumulation of certain proteins to the bud; binds to ER-derived membranes and targets mRNAs to cortical ER |
| *VPH2* | S000001602 | Integral membrane protein required for V-ATPase function; not an actual component of the vacuolar H+-ATPase (V-ATPase) complex; functions in the assembly of the V-ATPase; localized to the endoplasmic reticulum (ER); involved in methionine restriction extension of chronological lifespan in an autophagy-dependent manner |
| *DAN1* | S000003911 | Cell wall mannoprotein; has similarity to Tir1p, Tir2p, Tir3p, and Tir4p; expressed under anaerobic conditions, completely repressed during aerobic growth |
| *HOT13* | S000001567 | Zinc-binding mitochondrial intermembrane space (IMS) protein; involved in a disulfide relay system for IMS import of cysteine-containing proteins; binds Mia40p and stimulates its Erv1p-dependent oxidation, probably by sequestering zinc |
| *RRP14* | S000001565 | Essential protein, constituent of 66S pre-ribosomal particles; interacts with proteins involved in ribosomal biogenesis and cell polarity; member of the SURF-6 family |
| *TFA1* | S000001511 | TFIIE large subunit; involved in recruitment of RNA polymerase II to the promoter, activation of TFIIH, and promoter opening |
| *AQY2* | S000003975 | Water channel that mediates water transport across cell membranes; only expressed in proliferating cells; controlled by osmotic signals; may be involved in freeze tolerance; disrupted by a stop codon in many S. cerevisiae strains |
| *CMS1* | S000003993 | Putative subunit of the 90S preribosome processome complex; overexpression rescues supressor mutant of mcm10; null mutant is viable; relocalizes from nucleus to cytoplasm upon DNA replication stress |
| *TEN1* | S000004000 | Protein that regulates telomeric length; protects telomeric ends in a complex with Cdc13p and Stn1p; similar to human Ten1 which is critical for the telomeric function of the CST (Cdc13p-Stn1p-Ten1p) complex |
| *GAT3* | S000004003 | Protein containing GATA family zinc finger motifs; involved in spore wall assembly; sequence similarity to GAT4, and the double mutant gat3 gat4 exhibits reduced dityrosine fluorescence relative to the single mutants |
| *RIC1* | S000004029 | Protein involved in retrograde transport to the cis-Golgi network; forms heterodimer with Rgp1p that acts as a GTP exchange factor for Ypt6p; involved in transcription of rRNA and ribosomal protein genes |
| *CIS3* | S000003694 | Mannose-containing glycoprotein constituent of the cell wall; member of the PIR (proteins with internal repeats) family |
| *BUD25* | S000007590 | Protein involved in bud-site selection; diploid mutants display a random budding pattern instead of the wild-type bipolar pattern |
| *SMB1* | S000000831 | Core Sm protein Sm B; part of heteroheptameric complex |
| *CMC4* | S000028514 | Protein that localizes to the mitochondrial intermembrane space; localizes via the Mia40p-Erv1p system; contains twin cysteine-x(9)-cysteine motifs |
| *TDA1* | S000004905 | Protein kinase of unknown cellular role; green fluorescent protein (GFP)-fusion protein localizes to the cytoplasm and nucleus; null mutant is sensitive to expression of the top1-T722A allele; not an essential gene; relocalizes from nucleus to cytoplasm upon DNA replication stress |
| *CAT8* | S000004893 | Zinc cluster transcriptional activator; necessary for derepression of a variety of genes under non-fermentative growth conditions, active after diauxic shift, binds carbon source responsive elements; relative distribution to the nucleus increases upon DNA replication stress |
| *GOT1* | S000004906 | Homodimeric protein that is packaged into COPII vesicles; cycles between the ER and Golgi; involved in secretory transport but not directly required for aspects of transport assayed in vitro; may influence membrane composition |
| *FET4* | S000004938 | Low-affinity Fe(II) transporter of the plasma membrane |
| *KRE1* | S000005266 | Cell wall glycoprotein involved in beta-glucan assembly; serves as a K1 killer toxin membrane receptor |
| *BRE5* | S000005334 | Ubiquitin protease cofactor; forms deubiquitination complex with Ubp3p that coregulates anterograde and retrograde transport between the endoplasmic reticulum and Golgi compartments; null is sensitive to brefeldin A |
| *ESF2* | S000005337 | Essential nucleolar protein involved in pre-18S rRNA processing; binds to RNA and stimulates ATPase activity of Dbp8; involved in assembly of the small subunit (SSU) processome |
| *AIF1* | S000005357 | Mitochondrial cell death effector; translocates to the nucleus in response to apoptotic stimuli, homolog of mammalian Apoptosis-Inducing Factor, putative reductase |
| *BDS1* | S000005524 | Bacterially-derived sulfatase; required for use of alkyl- and aryl-sulfates as sulfur sources |
| *ENB1* | S000005518 | Endosomal ferric enterobactin transporter; expressed under conditions of iron deprivation; member of the major facilitator superfamily; expression is regulated by Rcs1p and affected by chloroquine treatment |
| *DCP1* | S000005509 | Subunit of the Dcp1p-Dcp2p decapping enzyme complex; decapping complex removes the 5' cap structure from mRNAs prior to their degradation; enhances the activity of catalytic subunit Dcp2p; regulated by DEAD box protein Dhh1p; forms cytoplasmic foci upon DNA replication stress |
| *ZEO1* | S000005469 | Peripheral membrane protein of the plasma membrane; interacts with Mid2p; regulates the cell integrity pathway mediated by Pkc1p and Slt2p; the authentic protein is detected in a phosphorylated state in highly purified mitochondria |
| *HAL9* | S000005449 | Putative transcription factor containing a zinc finger; overexpression increases salt tolerance through increased expression of the ENA1 (Na+/Li+ extrusion pump) gene while gene disruption decreases both salt tolerance and ENA1 expression |
| *RTG1* | S000005428 | Transcription factor (bHLH) involved in interorganelle communication; contributes to communication between mitochondria, peroxisomes, and nucleus |
| *MAM3* | S000005421 | Protein required for normal mitochondrial morphology; has similarity to hemolysins |
| *DDR2* | S000005413 | Multi-stress response protein; expression is activated by a variety of xenobiotic agents and environmental or physiological stresses |
| *GAL11* | S000005411 | Subunit of the RNA polymerase II mediator complex; associates with core polymerase subunits to form the RNA polymerase II holoenzyme; affects transcription by acting as target of activators and repressors; forms part of the tail domain of mediator |
| *FRE8* | S000004037 | Protein with sequence similarity to iron/copper reductases; involved in iron homeostasis; deletion mutant has iron deficiency/accumulation growth defects; expression increased in the absence of copper-responsive transcription factor Mac1p |
| *SPT8* | S000004045 | Subunit of the SAGA transcriptional regulatory complex; not present in SAGA-like complex SLIK/SALSA; required for SAGA-mediated inhibition at some promoters |
| *QRI5* | S000004194 | Mitochondrial inner membrane protein; required for accumulation of spliced COX1 mRNA; may have an additional role in translation of COX1 mRNA |
| *ENT2* | S000004196 | Epsin-like protein required for endocytosis and actin patch assembly; functionally redundant with Ent1p; contains clathrin-binding motif at C-terminus |
| *ARV1* | S000004232 | Cortical ER protein; implicated in the membrane insertion of tail-anchored C-terminal single transmembrane domain proteins; may function in transport of glycosylphosphatidylinositol intermediates into ER lumen; required for normal intracellular sterol distribution |
| *YPT6* | S000004252 | Rab family GTPase; Ras-like GTP binding protein involved in the secretory pathway, required for fusion of endosome-derived vesicles with the late Golgi, maturation of the vacuolar carboxypeptidase Y; resides temporarily at the Golgi, dissociates into cytosol upon arrival of the Rab GTPaseYpt32p, which also functions in the late Golgi; Golgi-localized form is bound to GTP, while cytosolic form is GDP-bound; homolog of the mammalian Rab6 |
| *OPI10* | S000005392 | Protein with a possible role in phospholipid biosynthesis; null mutant displays an inositol-excreting phenotype that is suppressed by exogenous choline; protein abundance increases in response to DNA replication stress |
| *SIN3* | S000005364 | Component of both the Rpd3S and Rpd3L histone deacetylase complexes; involved in transcriptional repression and activation of diverse processes, including mating-type switching and meiosis; involved in the maintenance of chromosomal integrity |
| *PFA4* | S000005363 | Palmitoyltransferase with autoacylation activity; required for palmitoylation of amino acid permeases containing a C-terminal Phe-Trp-Cys site; required for modification of Chs3p; member of the DHHC family of putative palmitoyltransferases |
| *RTS1* | S000005540 | B-type regulatory subunit of protein phosphatase 2A (PP2A); Rts1p and Cdc55p are alternative regulatory subunits for PP2A catalytic subunits, Pph21p and Pph22p; PP2A-Rts1p protects cohesin when recruited by Sgo1p to the pericentromere; highly enriched at centromeres in the absence of Cdc55p; required for maintenance of septin ring organization during cytokinesis, for ring disassembly in G1 and for dephosphorylation of septin, Shs1p |
| *SFM1* | S000005547 | SPOUT methyltransferase; catalyzes omega-monomethylation of Rps3p on Arg-146; not an essential gene; predicted to be involved in rRNA processing and ribosome biogenesis and in biopolymer catabolism |
| *CRS5* | S000005557 | Copper-binding metallothionein; required for wild-type copper resistance |
| *TOM6* | S000005571 | Component of the TOM (translocase of outer membrane) complex; responsible for recognition and initial import steps for all mitochondrially directed proteins; promotes assembly and stability of the TOM complex |
| *OST2* | S000005629 | Epsilon subunit of the oligosaccharyltransferase complex; located in the ER lumen; catalyzes asparagine-linked glycosylation of newly synthesized proteins |
| *OST4* | S000002391 | Subunit of the oligosaccharyltransferase complex of the ER lumen; complex catalyzes protein asparagine-linked glycosylation; type I membrane protein required for incorporation of Ost3p or Ost6p into the OST complex |
| *TOM5* | S000006433 | Component of the TOM (translocase of outer membrane) complex; responsible for recognition and initial import of all mitochondrially directed proteins; involved in transfer of precursors from the Tom70p and Tom20p receptors to the Tom40p pore |
| *RGS2* | S000005633 | Negative regulator of glucose-induced cAMP signaling; directly activates the GTPase activity of the heterotrimeric G protein alpha subunit Gpa2p |
| *RIO1* | S000005645 | Serine kinase involved in cell cycling and pre-rRNA processing; associated with late pre-40S particles via its conserved C-terminal domain and participates in late 40S biogenesis; association with pre-40S particles regulated by its catalytic ATPase site and likely occurs after the release of Rio2p from these particles; involved in cell cycle progression and processing of the 20S pre-rRNA into mature 18S rRNA; essential gene |
| *MED4* | S000005700 | Subunit of the RNA polymerase II mediator complex; associates with core polymerase subunits to form the RNA polymerase II holoenzyme; essential for transcriptional regulation |
| *FYV5* | S000000563 | Protein involved in regulation of the mating pathway; binds with Matalpha2p to promoters of haploid-specific genes; required for survival upon exposure to K1 killer toxin; involved in ion homeostasis |
| *RDL1* | S000005811 | Thiosulfate sulfurtransferase; contains a rhodanese-like domain; localized to the mitochondrial outer membrane; protein abundance increases in response to DNA replication stress; similar to the human TSTD gene |
| *FIT3* | S000005910 | Mannoprotein that is incorporated into the cell wall; incorporated via a glycosylphosphatidylinositol (GPI) anchor; involved in the retention of siderophore-iron in the cell wall |
| *CIN2* | S000006162 | GTPase-activating protein (GAP) for Cin4p; tubulin folding factor C involved in beta-tubulin (Tub2p) folding; mutants display increased chromosome loss and benomyl sensitivity; deletion complemented by human GAP, retinitis pigmentosa 2 |
| *CSM4* | S000006121 | Protein required for accurate chromosome segregation during meiosis; involved in meiotic telomere clustering (bouquet formation) and telomere-led rapid prophase movements; functions with meiosis-specific telomere-binding protein Ndj1p |
| *DAP1* | S000006091 | Heme-binding protein; involved in regulation of cytochrome P450 protein Erg11p; damage response protein, related to mammalian membrane progesterone receptors; mutations lead to defects in telomeres, mitochondria, and sterol synthesis |
| *REV3* | S000006088 | Catalytic subunit of DNA polymerase zeta; involved in translesion synthesis during post-replication repair; required for mutagenesis induced by DNA damage; involved in double-strand break repair |
| *SPO19* | S000006051 | Meiosis-specific prospore protein; required to produce bending force necessary for proper assembly of the prospore membrane during sporulation; identified as a weak high-copy suppressor of the spo1-1 ts mutation |
| *HHO1* | S000006048 | Histone H1, linker histone with roles in meiosis and sporulation; decreasing levels early in sporulation may promote meiosis, and increasing levels during sporulation facilitate compaction of spore chromatin; binds to promoters and within genes in mature spores; may be recruited by Ume6p to promoter regions, contributing to transcriptional repression outside of meiosis; suppresses DNA repair involving homologous recombination |
| *ERI1* | S000028423 | Endoplasmic reticulum membrane protein that binds and inhibits Ras2p; binds to and inhibits GTP-bound Ras2p at the endoplasmic reticulum (ER); component of the GPI-GnT complex which catalyzes the first step in GPI-anchor biosynthesis; probable homolog of mammalian PIG-Y protein |
| *MFM1* | S000005981 | Mitochondrial inner membrane magnesium transporter; involved in maintenance of mitochondrial magnesium concentrations and membrane potential; indirectly affects splicing of group II introns; functionally and structurally related to Mrs2p |
| *IRC15* | S000005938 | Microtubule associated protein; regulates microtubule dynamics; required for accurate meiotic chromosome segregation; null mutant displays large budded cells due to delayed mitotic progression, increased levels of spontaneous Rad52 foci |
| *ATG11* | S000006253 | Adapter protein for pexophagy and the Cvt targeting pathway; directs receptor-bound cargo to the phagophore assembly site (PAS) for packaging into vesicles; required for recruiting other proteins to the PAS; recruits Dnm1p to facilitate fission of mitochondria that are destined for removal by mitophagy |
| *ISR1* | S000006310 | Predicted protein kinase; overexpression causes sensitivity to staurosporine, which is a potent inhibitor of protein kinase C |
| *AXL1* | S000006326 | Haploid specific endoprotease of a-factor mating pheromone; performs one of two N-terminal cleavages during maturation of a-factor mating pheromone; required for axial budding pattern of haploid cells |
| *NUT2* | S000006372 | Subunit of the RNA polymerase II mediator complex; associates with core polymerase subunits to form the RNA polymerase II holoenzyme; required for transcriptional activation and has a role in basal transcription; protein abundance increases in response to DNA replication stress |
| *FAP7* | S000002325 | Essential NTPase required for small ribosome subunit synthesis; mediates processing of the 20S pre-rRNA at site D in the cytoplasm but associates only transiently with 43S preribosomes via Rps14p, may be the endonuclease for site D; depletion leads to accumulation of pre-40S ribosomes in 80S-like ribosomes |
| *CCT4* | S000002302 | Subunit of the cytosolic chaperonin Cct ring complex; related to Tcp1p, required for the assembly of actin and tubulins in vivo |
| *SCM3* | S000002298 | Nonhistone component of centromeric chromatin; binds to histone H3 variant, Cse4p, and recruits it to centromeres; involved in the assembly and maintenance of Cse4-H4 at centromeres; required for kinetochore assembly and G2/M progression; may protect Cse4p from ubiquitylation |
| *USO1* | S000002216 | Essential protein involved in vesicle-mediated ER to Golgi transport; binds membranes and functions during vesicle docking to the Golgi; required for assembly of the ER-to-Golgi SNARE complex |
| *PBP4* | S000002211 | Pbp1p binding protein; interacts strongly with Pab1p-binding protein 1 (Pbp1p) in the yeast two-hybrid system; also interacts with Lsm12p in a copurification assay; relative distribution to the nucleus increases upon DNA replication stress |
| *BSC1* | S000002195 | Protein of unconfirmed function; similar to cell surface flocculin Flo11p; ORF exhibits genomic organization compatible with a translational readthrough-dependent mode of expression |
| *MCD1* | S000002161 | Essential alpha-kleisin subunit of the cohesin complex; required for sister chromatid cohesion in mitosis and meiosis; apoptosis induces cleavage and translocation of a C-terminal fragment to mitochondria; expression peaks in S phase |
| *PSF1* | S000002420 | Subunit of the GINS complex (Sld5p, Psf1p, Psf2p, Psf3p); complex is localized to DNA replication origins and implicated in assembly of the DNA replication machinery |
| *GRX3* | S000002505 | Glutathione-dependent oxidoreductase; hydroperoxide and superoxide-radical responsive; monothiol glutaredoxin subfamily member along with Grx4p and Grx5p; protects cells from oxidative damage; with Grx4p, binds to Aft1p in iron-replete conditions, promoting its dissociation from promoters; evidence exists indicating that the translation start site is not Met1 as currently annotated, but rather Met36 |
| *CBS2* | S000002605 | Mitochondrial translational activator of the COB mRNA; interacts with translating ribosomes, acts on the COB mRNA 5'-untranslated leader |
| *SPC19* | S000002609 | Essential subunit of the Dam1 complex (aka DASH complex); complex couples kinetochores to the force produced by MT depolymerization thereby aiding in chromosome segregation |
| *IVY1* | S000002637 | Phospholipid-binding protein that interacts with both Ypt7p and Vps33p; may partially counteract the action of Vps33p and vice versa, localizes to the rim of the vacuole as cells approach stationary phase |
| *TRS23* | S000002654 | Core component of transport protein particle (TRAPP) complexes I-III; TRAPP complexes are related multimeric guanine nucleotide-exchange factor for the GTPase Ypt1p, regulating ER-Golgi traffic (TRAPPI), intra-Golgi traffic (TRAPPII), endosome-Golgi traffic (TRAPPII and III) and autophagy (TRAPPIII) |
| *HRQ1* | S000002699 | 3'-5' DNA helicase that has DNA strand annealing activity; helicase activity is stimulated by fork structure and 3'-tail length of substrates; acts with Rad4p in nucleotide-excision repair; belongs to the widely conserved RecQ family of proteins which are involved in maintaining genomic integrity; similar to the human RecQ4p implicated in Rothmund-Thomson syndrome (RTS) |
| *HNT2* | S000002713 | Dinucleoside triphosphate hydrolase; has similarity to the tumor suppressor FHIT and belongs to the histidine triad (HIT) superfamily of nucleotide-binding proteins |
| *TFB1* | S000002719 | Subunit of TFIIH and nucleotide excision repair factor 3 complexes; required for nucleotide excision repair, target for transcriptional activators; relocalizes to the cytosol in response to hypoxia |
| *YCG1* | S000002733 | Subunit of the condensin complex; required for establishment and maintenance of chromosome condensation, chromosome segregation and chromatin binding of the condensin complex; required for clustering of tRNA genes at the nucleolus; required for replication slow zone (RSZ) breakage following Mec1p inactivation |
| *RQC1* | S000002741 | Component of the ribosome quality control complex (RQC); RQC (Rqc1p-Rkr1p-Tae2p-Cdc48p-Npl4p-Ufd1p) is a ribosome-bound complex required for the degradation of polypeptides arising from stalled translation; required along with Rkr1p for recruitment of the Cdc48p-Npl4p-Ufd1p AAA ATPase complex to the RQC |
| *BCP1* | S000002769 | Essential protein involved in nuclear export of Mss4p; Mss4p is a lipid kinase that generates phosphatidylinositol 4,5-biphosphate and plays a role in actin cytoskeleton organization and vesicular transport |
| *KEI1* | S000002775 | Component of inositol phosphorylceramide (IPC) synthase; forms a complex with Aur1p and regulates its activity; required for IPC synthase complex localization to the Golgi; post-translationally processed by Kex2p; KEI1 is an essential gene |
| *STE14* | S000002818 | Farnesyl cysteine-carboxyl methyltransferase; mediates the carboxyl methylation step during C-terminal CAAX motif processing of a-factor and RAS proteins in the endoplasmic reticulum, localizes to the ER membrane |
| *NPL3* | S000002840 | RNA-binding protein; promotes elongation, regulates termination, and carries poly(A) mRNA from nucleus to cytoplasm; represses translation initiation by binding eIF4G; required for pre-mRNA splicing; interacts with E3 ubiquitin ligase Bre1p, linking histone ubiquitination to mRNA processing; may have role in telomere maintenance; dissociation from mRNAs promoted by Mtr10p; phosphorylated by Sky1p in cytoplasm; protein abundance increases in response to DNA replication stress |
| *SPG3* | S000002912 | Protein required for high temperature survival during stationary phase; not required for growth on nonfermentable carbon sources |
| *AGP3* | S000001839 | Low-affinity amino acid permease; may act to supply the cell with amino acids as nitrogen source in nitrogen-poor conditions; transcription is induced under conditions of sulfur limitation; plays a role in regulating Ty1 transposition |
| *DAK2* | S000001841 | Dihydroxyacetone kinase; required for detoxification of dihydroxyacetone (DHA); involved in stress adaptation |
| *AUA1* | S000001955 | Protein required for the negative regulation by ammonia of Gap1p; Gap1p is a general amino acid permease |
| *KEG1* | S000001938 | Integral membrane protein of the ER; physically interacts with Kre6p; has a role in the synthesis of beta-1,6-glucan in the cell wall; required for cell viability |
| *NSE4* | S000002263 | Component of the SMC5-SMC6 complex; this complex plays a key role in the removal of X-shaped DNA structures that arise between sister chromatids during DNA replication and repair |
| *NKP2* | S000004307 | Central kinetochore protein and subunit of the Ctf19 complex; mutants have elevated rates of chromosome loss; orthologous to fission yeast kinetochore protein cnl2 |
| *MMS22* | S000004312 | Subunit of E3 ubiquitin ligase complex involved in replication repair; stabilizes protein components of the replication fork, such as the fork-pausing complex and leading strand polymerase, preventing fork collapse and promoting efficient recovery during replication stress; required for accurate meiotic chromosome segregation |
| *REC102* | S000004321 | Protein involved in early stages of meiotic recombination; required for chromosome synapsis; forms a complex with Rec104p and Spo11p necessary during the initiation of recombination |
| *PSY3* | S000004368 | Component of Shu complex (aka PCSS complex); Shu complex also includes Shu1, Csm2, Shu2, and promotes error-free DNA repair; promotes Rad51p filament assembly; Shu complex mediates inhibition of Srs2p function; Psy3p and Csm2p contain similar DNA-binding regions which work together to form a single DNA binding site; deletion of PSY3 results in a mutator phenotype; deletion increases sensitivity to anticancer drugs oxaliplatin and cisplatin but not mitomycin C |
| *TSR2* | S000004427 | Protein with a potential role in pre-rRNA processing |
| *PSP2* | S000004479 | Asn rich cytoplasmic protein that contains RGG motifs; high-copy suppressor of group II intron-splicing defects of a mutation in MRS2 and of a conditional mutation in POL1 (DNA polymerase alpha); possible role in mitochondrial mRNA splicing |
| *MIH1* | S000004639 | Protein tyrosine phosphatase involved in cell cycle control; regulates the phosphorylation state of Cdc28p |
| *SEN15* | S000004663 | Subunit of the tRNA splicing endonuclease |
| *PSO2* | S000004745 | Nuclease required for DNA single- and double-strand break repair; acts at a post-incision step in repair of breaks that result from interstrand cross-links produced by a variety of mono- and bi-functional psoralen derivatives; induced by UV-irradiation; forms nuclear foci upon DNA replication stress |
| *TPP1* | S000004765 | DNA 3'-phosphatase; functions in repair of endogenous damage of double-stranded DNA, activity is specific for removal of 3' phosphates at strand breaks; similar to the l-2-haloacid dehalogenase superfamily; homolog of human polynucleotide kinase/3′-phosphatase |
| *HLJ1* | S000004771 | Co-chaperone for Hsp40p; anchored in the ER membrane; with its homolog Ydj1p promotes ER-associated protein degradation (ERAD) of integral membrane substrates |
| *PET309* | S000004057 | Specific translational activator for the COX1 mRNA; binds to the COX1 mRNA; also influences stability of intron-containing COX1 primary transcripts; localizes to the mitochondrial inner membrane; contains 12 pentatricopeptide repeats |
| *BOS1* | S000004068 | v-SNARE (vesicle specific SNAP receptor); localized to the endoplasmic reticulum membrane and necessary for vesicular transport from the ER to the Golgi; required for efficient nuclear fusion during mating |
| *MIM2* | S000007618 | Mitochondrial protein required for outer membrane protein import; involved in import of the subset of proteins with multiple alpha-helical transmembrane segments, including Ugo1p, Tom20p, and Fzo1p; component of a large protein complex in the outer membrane that includes Mim1p; not essential in W303 strain background |
| *CCW12* | S000004100 | Cell wall mannoprotein; plays a role in maintenance of newly synthesized areas of cell wall; localizes to periphery of small buds, septum region of larger buds, and shmoo tip |
| *APS1* | S000004160 | Small subunit of the clathrin-associated adaptor complex AP-1; AP-1 is involved in protein sorting at the trans-Golgi network; homolog of the sigma subunit of the mammalian clathrin AP-1 complex |
| *HCR1* | S000004182 | eIF3j component of translation initiation factor 3 (eIF3); dual function protein involved in translation initiation as a substoichiometric component (eIF3j) of eIF3; required for processing of 20S pre-rRNA; required at post-transcriptional step for efficient retrotransposition; absence results in decreased Ty1 Gag:GFP protein levels; binds to eIF3 subunits Rpg1p and Prt1p and 18S rRNA |
| *PDR3* | S000000101 | Transcriptional activator of the pleiotropic drug resistance network; regulates expression of ATP-binding cassette (ABC) transporters through binding to cis-acting PDRE sites (PDR responsive elements); has a role in response to drugs and organic solvents; post-translationally up-regulated in cells lacking functional mitochondrial genome; involved in diauxic shift; relative distribution to nucleus increases upon DNA replication stress; APCC(Cdh1) substrate |
| *TAE1* | S000000465 | AdoMet-dependent proline methyltransferase; catalyzes the dimethylation of ribosomal proteins Rpl12 and Rps25 at N-terminal proline residues; has a role in protein synthesis; fusion protein localizes to the cytoplasm |
| *VBA2* | S000000497 | Permease of basic amino acids in the vacuolar membrane |
| *ADF1* | S000028518 | Transcriptional repressor encoded by the FYV5 antisense strand; negatively regulates transcription of FYV5 by binding to the promoter on the sense strand |
| *SGF29* | S000000516 | Component of the HAT/Core module of the SAGA, SLIK, and ADA complexes; HAT/Core module also contains Gcn5p, Ngg1p, and Ada2p; binds methylated histone H3K4; involved in transcriptional regulation through SAGA and TBP recruitment to target promoters and H3 acetylation |
| *RER1* | S000000507 | Protein involved in retention of membrane proteins; including Sec12p, in the ER; localized to Golgi; functions as a retrieval receptor in returning membrane proteins to the ER |
| *SRD1* | S000000611 | Protein involved in the processing of pre-rRNA to mature rRNA; contains a C2/C2 zinc finger motif; srd1 mutation suppresses defects caused by the rrp1-1 mutation |
| *SOM1* | S000002954 | Subunit of the mitochondrial inner membrane peptidase (IMP); IMP is required for maturation of mitochondrial proteins of the intermembrane space; Som1p facilitates cleavage of a subset of substrates; contains twin cysteine-x9-cysteine motifs |
| *ERG28* | S000000846 | Endoplasmic reticulum membrane protein; may facilitate protein-protein interactions between the Erg26p dehydrogenase and the Erg27p 3-ketoreductase and/or tether these enzymes to the ER, also interacts with Erg6p |
| *TCA17* | S000000774 | Component of transport protein particle (TRAPP) complex II; TRAPPII is a multimeric guanine nucleotide-exchange factor for the GTPase Ypt1p, regulating intra-Golgi and endosome-Golgi traffic; promotes association of TRAPPII-specific subunits with the TRAPP core complex; sedlin related; human Sedlin mutations cause SEDT, a skeletal disorder |

**Additional Figures**

**Figure S1.** Statistical analysis on the number of contigs with identity ＜95% in the amino acid levels

**Figure S2.** Analysis of two *BIO* genes present in the LI genome, but not in the S288c genome. A: Schematic organization of two relevant segments from MT1-contig40, S288c-Chr.IX and K7-Chr.IX; B: Dot Matrix Comparison maps of the DNA sequences between *BIO1*-MT1/*BIO6*-MT1 and *BIO1*-Ref/*BIO6*-Ref; C:Dot Matrix Comparison maps of the protein sequences between *BIO1*-MT1/*BIO6*-MT1 and *BIO1*-Ref/*BIO6*-Ref.

**Figure S3.** A: Colinearity analysis of Chr. I. The same Locally Collinear Block is represented by same color and vertical lines represent the same LCB; The height of internal vertical lines in LCB on behalf of the level of sequence consistency; Blank in one strain stands for the region does not exist in the other. B: PCR profiles of the [25Kb](app:ds:deletion) fragment specific to S288c which was divided into 4 small [segment](app:ds:fragment)s (S1, S2, S3, S4). In addition, 26S rDNA gene was detected as the positive control. M: DNA maker of 5000bp.
